# Assembling the Formulation Puzzle: Comparative Evaluation of Biobased and Conventional Microemulsions for Enhanced Oil Recovery

**DOI:** 10.1021/acsomega.5c12752

**Published:** 2026-04-07

**Authors:** Verena F. B. Santos, Adriana V. Santos, Pamela D. Rodrigues, Flávia C. C. Santos, Aline O. Santos, Victoria S. Souza, Landson S. Marques, Cristina M. Quintella, George Simonelli, Luiz Carlos L. Santos

**Affiliations:** † Oil, Gas, and Biofuels Research Laboratory (PGBio), Postgraduate Program in Chemical Engineering (PPEQ), 28111 Federal University of Bahia (UFBA), Rua Prof. Aristides Novis 02, Federação, Salvador, Bahia 40210-630, Brazil; ‡ Institute and Center for Energy and Environment (CIENAM), Federal University of Bahia (UFBA). Av. Adhemar de Barros, s/n, 2° andar, Ondina, Salvador, Bahia 40301-110, Brazil; § Federal Institute of Education, Science and Technology of Bahia (IFBA), Advanced Energy Research and Study Group (GEPAE), São Cristóvão, Itinga, Lauro de Freitas, Bahia 42739-005, Brazil; ∥ Postgraduate Program in Geochemistry: Petroleum and Environment (POSPETRO), Federal University of Bahia (UFBA). Av. Adhemar de Barros, s/n, 2° andar, Ondina, Salvador, Bahia 40301-110, Brazil

## Abstract

The rational design of environmentally friendly microemulsions for chemical enhanced oil recovery (EOR) remains a critical challenge, particularly in achieving interfacial efficiency and thermodynamic stability comparable to conventional petrochemical systems. In this study, we deconstruct the formulation puzzle by systematically comparing eight Winsor IV microemulsions composed of renewable and conventional components under reservoir-relevant conditions (60 °C). The systems were engineered using different combinations of nonpolar phases (toluene or pine oil), surfactants (Triton X-100 or saponified coconut oil), cosurfactants (isopropyl or ethyl alcohol), and polar phases (distilled water, saline brine, or glycerol), to reveal how molecular constituents cooperatively dictate phase architecture and physicochemical properties. Biobased formulations consistently produced significantly broader Winsor IV domains, up to 35% larger than their petrochemical analogues, while achieving low interfacial tensions (0.14–2.7 mN·m^–1^) and moderate viscosities (≤4.13 cP), effectively balancing injectivity with capillary desaturation potential. The synergistic pairing of saponified coconut oil with short-chain alcohols yielded flexible and resilient interfacial films, enabling enhanced performance across thermal and compositional gradients. For the first time, this work demonstrates that renewable surfactant–cosurfactant frameworks not only rival but, under the investigated thermal and salinity conditions, can surpass traditional hydrocarbon-based systems in interfacial behavior and formulation robustness, establishing a scalable and sustainable foundation for next-generation EOR applications.

## Graphical abstract

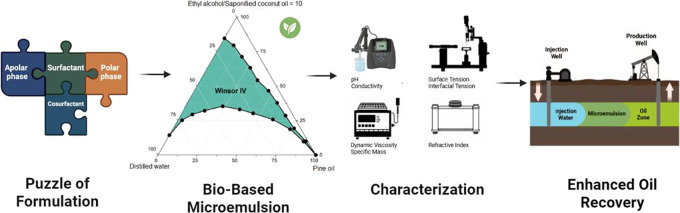

## 1 Introduction

The recovery of large volumes of residual oil after primary and secondary stages remains a central challenge in the oil and gas industry.^1,2^ Conventional methods, such as extraction by natural pressure or water and gas injection, recover only a fraction of the original oil in place (OOIP), making enhanced oil recovery (EOR) techniques indispensable.^3,4^ Among these, chemical methods, including polymers,^5^ surfactants,^6^ alkalis,^7^ and microemulsions,^8,9^ stand out as promising solutions to mobilize residual oil.

Microemulsions are particularly attractive for EOR due to their ability to form single-phase, isotropic, and thermodynamically stable systems when polar and nonpolar phases, surfactants, and cosurfactants are appropriately balanced.^10^ This formulation process can be viewed as a “puzzle,” in which the synergistic interaction of all components governs interfacial tension (IFT) reduction, wettability alteration, and oil mobilization.^11^ Within this framework, Winsor IV microemulsions are of special interest, as their single-phase nature, compositional flexibility, and tolerance to salinity and temperature variations make them well suited for reservoir applications.^10^

However, conventional microemulsion systems based on petrochemical surfactants face well-documented limitations, including environmental persistence, toxicity, and dependence on nonrenewable feedstocks. In the context of chemical EOR, environmental friendliness should not be interpreted solely as the renewable origin of formulation components, but rather as their functional biocompatibility under reservoir and postproduction conditions. Environmentally friendly formulations are defined by their ability to exhibit low aquatic toxicity, accelerated biodegradability, and reduced environmental persistence after breakthrough or during produced-water treatment, while maintaining effective interfacial performance under harsh salinity and temperature conditions.^12,13^ Operationally, such fluids mitigate risks associated with surface handling and subsurface contamination, without requiring toxic cosolvents to stabilize microemulsion structures.^13^

Recent studies highlight that these formulations generate effluents requiring complex downstream treatment and are increasingly constrained by emerging environmental regulations.^14^ As a result, there is growing pressure for EOR technologies to align with internationally recognized sustainability frameworks, particularly the United Nations Sustainable Development Goals (SDGs). The search for biobased surfactants and nonpolar phases is therefore not only technically advantageous but also aligned with regulatory and societal drivers for sustainability.^14,15^ Within EOR applications, sustainability must be understood as a life-cycle concept integrating renewable sourcing, technical efficiency, and economic viability. Sustainable formulations are those capable of mobilizing residual oil with efficiency comparable or superior to synthetic systems, while minimizing chemical losses, adsorption on reservoir rock, and environmental footprint.^13,16,17^ Excessive surfactant adsorption (>1 mg·g^–1^) compromises both economic and environmental sustainability, making the incorporation of adsorption-mitigation strategies, such as sacrificial agents or nanoparticles, a critical design requirement.^18,19^

Assembling this puzzle requires a precise balance of molecular interactions, adjusting hydrophilic and lipophilic forces to optimize critical physicochemical properties such as low IFT, moderate viscosity, and thermal and salinity stability.^11,20,21^ Formulation robustness, in this context, refers to the physicochemical resilience of microemulsion systems to compositional dilution, salinity gradients, ion exchange with reservoir mineralogy, and temperature variations inherent to heterogeneous porous media. Robust formulations preserve their structural integrity, ideally maintaining a single-phase Winsor IV system or a middle-phase Winsor III microemulsion with ultralow IFT, while resisting premature phase separation and rheological instability, even in the presence of divalent ions or elevated temperatures.^11,22,23^

Beyond compositional stability, the practical significance of expanded Winsor IV domains is directly associated with the preservation of phase integrity and its impact on pore-scale oil displacement mechanisms under dynamic flow conditions. The literature consistently demonstrates that broader single-phase Winsor IV regions increase the likelihood of generating and sustaining microemulsions in situ during injection, even in the presence of dilution by formation water, salinity gradients, and thermal fluctuations characteristic of reservoir environments.^11,22^ This in situ persistence of a homogeneous microemulsion phase is critical to maintaining formulation efficiency throughout propagation in porous media.

From a capillary perspective, the stability of Winsor IV systems ensures the continuous presence of ultralow interfacial tension, which is essential for enhancing the capillary number and mobilizing trapped oil ganglia. The resulting reduction in capillary forces facilitates capillary desaturation, suppresses snap-off events in pore throats, and promotes improved pore connectivity, thereby enabling continuous oil displacement under flow.^19,22,24^ Notably, recent studies have shown that single-phase, dilutable microemulsions formulated with mixed surfactants can traverse bicontinuous microstructures while preserving ultralow IFT during progressive dilution, a prerequisite for sustained oil mobilization in realistic reservoir conditions.^22^

Equally important, the absence of premature phase separation in Winsor IV systems prevents the formation of viscous macroemulsions or multiphase fronts near the wellbore, which are known to impair injectivity and destabilize displacement fronts.^8,21^ Instead, homogeneous microemulsions propagate uniformly through the pore network, maximizing fluid–oil contact and minimizing preferential flow paths and bypassing.^25,26^ In addition, the controlled rheological behavior of these systems, often exhibiting pseudoplastic characteristics, supports high injectivity at elevated shear rates near the wellbore while maintaining sufficient viscosity for mobility control away from the well.^16,27^

Consequently, formulations characterized by broader Winsor IV domains translate favorable phase behavior into incremental oil recovery under realistic flow conditions. The combined effects of spontaneous solubilization, sustained ultralow IFT, wettability alteration toward more water-wet states, and stable transport through porous media underpin the superior performance of single-phase microemulsions in tertiary recovery, overcoming the transport limitations typically associated with unstable or multiphase systems (Winsor I or II).^17,28^

The validation of biobased microemulsions for EOR requires a mechanistic framework that extends beyond the renewable origin of formulation components, anchoring performance on interfacial robustness under thermal stress and molecular-level control of transport phenomena in porous media. Recent advances demonstrate that precisely engineered green systems can rival, and in some cases surpass, the stability and efficiency of conventional synthetic surfactants when designed on a sound physicochemical basis.

From a thermal robustness perspective, stability under elevated temperatures remains a prerequisite for applications in deep reservoirs and hybrid thermal–chemical recovery processes. Saw et al.^27^ established an important benchmark by formulating green nanoemulsions based on methyl esters derived from edible oils combined with nonionic surfactants (Tween 40/80). These systems exhibited pseudoplastic behavior and robust kinetic stability at temperatures up to 343 K, leading to a pronounced reduction in heavy oil viscosity and interfacial tension. The results demonstrate that environmental compatibility can coexist with the thermal resistance required for mobilizing highly viscous oils. Complementarily, de Oliveira et al.^29^ validated the synergistic coupling of microemulsions with steam injection, showing that reduced surface tension (23–25 mN·m^–1^) and wettability alteration enabled efficient heavy oil recovery by overcoming mass transfer limitations typically associated with standalone thermal methods.

At the molecular scale, displacement efficiency is governed by interfacial transport dynamics at the fluid–rock interface. Xiao et al.,^22^ elucidated phase transition mechanisms in single-phase microemulsion systems through molecular dynamics simulations, demonstrating that the interfacial distribution of mixed cationic/nonionic surfactants and electrostatic interactions control microstructure organization, conductive channel formation, and emulsion stability. These insights provide a theoretical foundation for designing formulations capable of maintaining structural integrity during dilution and propagation within the reservoir.

Wettability control further emerges as a decisive mechanism linking interfacial science to recovery efficiency. Tliba et al.^30^ demonstrated that surface functionalization of silica nanoparticles induces extreme wettability alteration in sandstone, with contact angles increasing from ∼20 to 173°. As the measurements were performed through the oil phase, this shift indicates a transition from oil-wet conditions (oil spreading) to strongly water-wet behavior (oil repellence). This behavior, driven by structural disjoining pressure and electrostatic interactions at the three-phase contact line, activated spontaneous imbibition processes and resulted in oil recoveries exceeding 77%, highlighting the role of nanostructured additives in enhancing fluid transport under confinement.

From a sustainability standpoint, effectiveness must be justified by clear physicochemical arguments rather than broad environmental claims. Mumbere et al.^31^ demonstrated that synergistic integration of low-salinity water flooding and nanoparticles mitigates surfactant adsorption and particle agglomeration, two of the most critical economic and technical challenges in chemical EOR. In parallel, the ability of ester-based microemulsions, such as those reported by Saw et al.,^27^ to act as biodegradable oil phases eliminates reliance on petrochemical hydrocarbons (e.g., *n*-heptane or diesel), offering thermodynamically stable systems with reduced toxicity and environmental persistence.

Collectively, these studies demonstrate that the transition toward green microemulsions is not driven solely by sustainability imperatives, but by robust experimental and theoretical evidence showing that renewable and hybrid molecular architectures can effectively manipulate complex interfacial phenomena and withstand extreme reservoir conditions, as established by recent advances in colloid and interface science.

The polar phase, typically water or brine, influences phase behavior, with salinity playing a key role in stability. The nonpolar phase, traditionally composed of hydrocarbons such as toluene, can be replaced by sustainable alternatives such as pine oil, which offers lower toxicity and greater environmental compatibility.^22^ The surfactant, essential for reducing IFT, may include biobased options such as saponified coconut oil, bringing ecological and economic benefits,^3^ while the cosurfactant, such as isopropanol, adjusts interfacial curvature to promote single-phase formation. Additives such as glycerol or nanoparticles may enhance stability and wettability.^3^

Detailed characterization of these properties is fundamental for evaluating microemulsion performance under reservoir conditions, including pH (7–10), conductivity (0.1–5 μS·cm^–1^), and tolerance to high temperatures.^21^ Despite their potential, biobased formulations have predominantly yielded Winsor I or III microemulsions, leaving the scope of sustainable Winsor IV systems underexplored.^4,23^ Studies such as those by Ferreira et al.,^32^ Jeirani et al.,^33^ and Pal et al.,^16^ investigated microemulsions with biobased components, focusing on isolated phases and analyzing properties under realistic conditions, but systematic comparisons between conventional and biobased Winsor IV systems remain scarce.

This study proposes the design and comparative evaluation of Winsor IV formulations using both conventional and biobased components. Through the metaphor of the “formulation puzzle,” we explore how the synergistic adjustment of each component influences stability and adaptability, highlighting the potential of systems containing pine oil and saponified coconut oil to offer sustainable and efficient solutions for EOR.

Although numerous studies have investigated microemulsion systems for chemical enhanced oil recovery, most reports focus on isolated formulation classes, incremental compositional changes, or performance optimization under a limited set of conditions. In contrast, the present work introduces a formulation-level and comparative framework that systematically evaluates renewable and petrochemical Winsor IV microemulsions under identical and reservoir-relevant conditions.

The novelty of this study lies in the direct side-by-side benchmarking of biobased and conventional microemulsions, integrating phase behavior mapping, interfacial tension, rheological response, salinity tolerance, and thermal stability within a unified experimental design. Moreover, this work demonstrates that wide and robust Winsor IV domains, combined with low interfacial tension and favorable rheology, can be achieved using fully biobased oil and surfactant systems without reliance on polymers, alkali, or nanoparticles.

By explicitly linking molecular-level formulation design to phase robustness and displacement-relevant properties, this study provides new insights into performance trade-offs and operational flexibility that are not addressed in previous microemulsion EOR studies, thereby advancing the rational design of sustainable chemical EOR formulations.

In this context, the present study aims to systematically design and comparatively evaluate renewable and petrochemical Winsor IV microemulsion systems under reservoir-relevant conditions (60 °C). Specifically, the objectives are to: (i) investigate the influence of formulation components on phase behavior and the extension of Winsor IV regions; (ii) evaluate key physicochemical properties, including interfacial tension, viscosity, density, and conductivity; and (iii) establish mechanistic links between formulation robustness, interfacial efficiency, and the expected performance of microemulsions in EOR applications.

## 2 Materials and Methods

### 2.1 Materials

The biobased anionic surfactant was synthesized through the saponification of coconut oil. The conventional surfactant employed was Triton X-100 (laboratory grade, Sigma-Aldrich). The nonpolar phases consisted of pine oil (commercial grade) and toluene (Cromoline, 99.5%). Cosurfactants included isopropyl alcohol (Synth, 99.5%) and ethyl alcohol (Êxodo Científica, 99.8%). Polar phases were prepared using distilled water, NaCl solutions (1 and 2% w/v), and glycerol (Synth, 99.5%).

### 2.2 Experimental Design and the Logic of the “Puzzle”

The experimental design was based on the “formulation puzzle” analogy (Figure 1), in which a systematic two-by-two combination of different components, while keeping the others constant, enabled the identification of synergistic interactions that influence the properties of the microemulsified systems.

**Figure 1:**
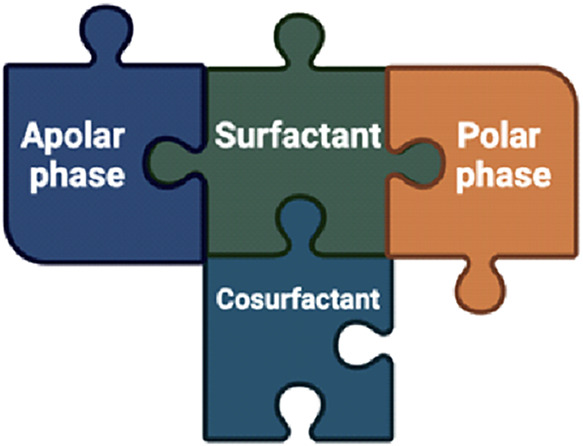
Conceptual representation using a puzzle analogy to illustrate the cooperative role of each component in stabilizing Winsor IV microemulsions for the EOR.

This strategy yielded eight distinct formulations (F1–F8); five formulations were biobased, produced from saponified coconut oil with pine oil, while two were conventional, composed of Triton X-100 with toluene or saponified coconut oil with toluene. Each surfactant–oil pair (S–A) was combined with one of the two alcohols and one of the two polar phases (C–P), generating the complete formulation set. This approach enabled the identification of the most promising formulations based on both technical performance and environmental considerations. The detailed composition of each formulation is presented in Table 1, providing the foundation for microemulsion preparation and subsequent phase diagram construction.

**Table 1:** Composition of Formulations F1–F8 for Microemulsion Preparation, Including the Nonpolar Phase, Surfactant, Cosurfactant, and Polar Phase^a^

**code**	**nonpolar phase**	**surfactant**	**cosurfactant**	**polar phase**
**F1**	pine oil	saponified coconut oil	isopropyl alcohol	distilled water
**F2**	pine oil	saponified coconut oil	ethyl alcohol	distilled water
**F3**	pine oil	saponified coconut oil	isopropyl alcohol	glycerol
**F4**	toluene	saponified coconut oil	isopropyl alcohol	distilled water
**F5**	pine oil	Triton X-100	isopropyl alcohol	distilled water
**F6**	pine oil	saponified coconut oil	isopropyl alcohol	saline water (1% w/v NaCl)
**F7**	pine oil	saponified coconut oil	isopropyl alcohol	saline water (2% w/v NaCl)
**F8**	toluene	Triton X-100	isopropyl alcohol	distilled water

a Note: For all formulations, the cosurfactant/surfactant (C/S) mass ratio was kept constant at 10.

Microemulsions were prepared using the mass titration method to determine the single-phase Winsor IV regions. Preweighed mixtures of the apolar phase (A) and cosurfactant/surfactant blend (C/S) were placed in test tubes, maintaining a fixed mass ratio of C/S = 10. The polar phase (P) was gradually added dropwise under constant magnetic stirring (IKA C-MAG HS) until the phase inversion point was reached, identified by a visual transition from turbid to transparent, indicating the formation of a single-phase microemulsion. The exact composition of each point was determined by mass balance using an analytical balance (Shimadzu AUY220).

The compositional data were normalized to mass fractions and plotted using OriginPro 2025 for the construction of the pseudoternary phase diagrams. The single-phase Winsor IV regions were delineated based on experimentally identified transition points, and their areas were calculated by numerical integration in OriginPro 2025. The areas are expressed in normalized arbitrary units (u^2^), representing the fraction of the total area of the pseudoternary diagram, which reflects the robustness of the thermodynamic stability of the formulations. Each diagram was constructed in triplicate to ensure reproducibility, with calculated standard deviations of the areas below 2%.

The crude oil used in the IFT experiments was supplied by PetroRecôncavo and originates from the Pojuca field, located in the Recôncavo Baiano Basin, Brazil. Its physicochemical properties are summarized in Table 2.

**Table 2:** Analyzed Physicochemical Properties of Crude Oil from the Pojuca Field, Recôncavo Baiano, Brazil

**physicochemical properties**	**defined value**	**technical standard**
pour point (°C)	34	ASTM D97
viscosity at 60 °C (cP)	17.1	ASTM D445
specific gravity at 20 °C (g·cm^–3^)	0.8824	ASTM D4052
specific gravity at 60 °C (g·cm^–3^)	0.8544	ASTM D4052
TAN (mg/KOH)	0.06	ASTM 974
BSW (%)	10	NBR-14647
saturated (%)	37.1	ASTM D3279 and ASTM D2007
aromatics (%)	28.1	ASTM D3279 and ASTM D2007
resins (%)	16.4	ASTM D3279 and ASTM D2007
asphaltenes (%)	5.2	ASTM D3279 and ASTM D2007
API grade	28.8	ASTM D4052

### 2.3 Analysis of the Physicochemical Properties of Microemulsions

For comparative characterization, a specific point within the Winsor IV region of each of the eight systems was selected, with a fixed composition of 60% C/S, 30% polar (P) phase, and 10% nonpolar (A) phase. All analyses were conducted in triplicate at 60 °C. Dynamic viscosity and specific gravity were measured using an automated viscometer and densimeter (Anton Paar SVM 3000). Electrical conductivity and pH were determined with a conductivity meter (Tecnal TEC-4MP) and a pH meter (MS Tecnopon), respectively. The refractive index was measured using a benchtop refractometer (Anton Paar Abbemat RXA). Surface tension (air/microemulsion) and IFT (oil/microemulsion) were evaluated using a tensiometer (DataPhysics OCA 15 plus) via the hanging drop method.

## 3 Results and Discussion

### 3.1 Single-Phase Stability Regions (Winsor IV) in Pseudoternary Diagrams

The formulation strategy adopted in this work enables a direct and formulation-level assessment of the relative advantages and limitations of renewable and petrochemical Winsor IV microemulsions, a perspective that, to the best of our knowledge, is not explicitly addressed in the existing literature.

This study establishes a comparative framework specifically tailored to intermediate-temperature reservoirs (∼60 °C), representative of shallow siliciclastic and mid-depth carbonate formations in the Recôncavo Basin, Bahia, Brazil. By conceptualizing the formulation process as a “puzzle assembly”, we provide a novel lens to dissect the molecular synergies among the nonpolar phase (pine oil or toluene), surfactant (saponified coconut oil or Triton X-100), cosurfactant (isopropyl or ethyl alcohol), and polar phase (distilled water, saline solutions, or glycerol). This metaphor not only elucidates the intricate interplay of components but also highlights our innovative integration of underexplored biobased materials, pine oil (a terpene-rich, biodegradable nonpolar phase), and saponified coconut oil (a renewable anionic surfactant) - into Winsor IV systems, which have been largely overlooked in prior EOR research favoring conventional, petroleum-derived formulations.^4,23^ Unlike existing studies that focus on isolated biobased components or Winsor I/III phases,^32–34^ our work fills a critical void by demonstrating that biobased Winsor IV microemulsions can outperform conventional ones in phase stability, interfacial efficiency, and environmental sustainability, paving the way for greener EOR strategies in real-world reservoirs.

All eight formulations (F1–F8) achieved thermodynamically stable Winsor IV single-phase microemulsions across defined composition ranges, a foundational achievement that underscores their EOR viability. Importantly, the biobased systems (F1–F3, F6–F7) exhibited markedly superior robustness, with expanded Winsor IV regions, low IFT, tunable viscosities for injectability, and enhanced biodegradability, attributes that address the longstanding challenge of balancing performance with eco-friendliness in chemical EOR.^2,3^ This novelty stems from our systematic substitution of toxic conventional components (e.g., toluene and Triton X-100) with renewable alternatives, revealing unprecedented synergies that enable Winsor IV stability under saline and glycerol-modified conditions, scenarios rarely explored for biobased systems.

To quantify this stability, we calculated the areas of the single-phase regions through numerical integration of pseudoternary phase diagrams (constructed as detailed in Section 2.2), expressed in normalized arbitrary units (u^2^). This metric innovatively captures the formulations’ tolerance to compositional fluctuations, a key advantage for heterogeneous reservoirs where precise control is impractical. The results, presented in the bar graph of Figure 2, reveal significant variations driven by component interactions.

**Figure 2:**
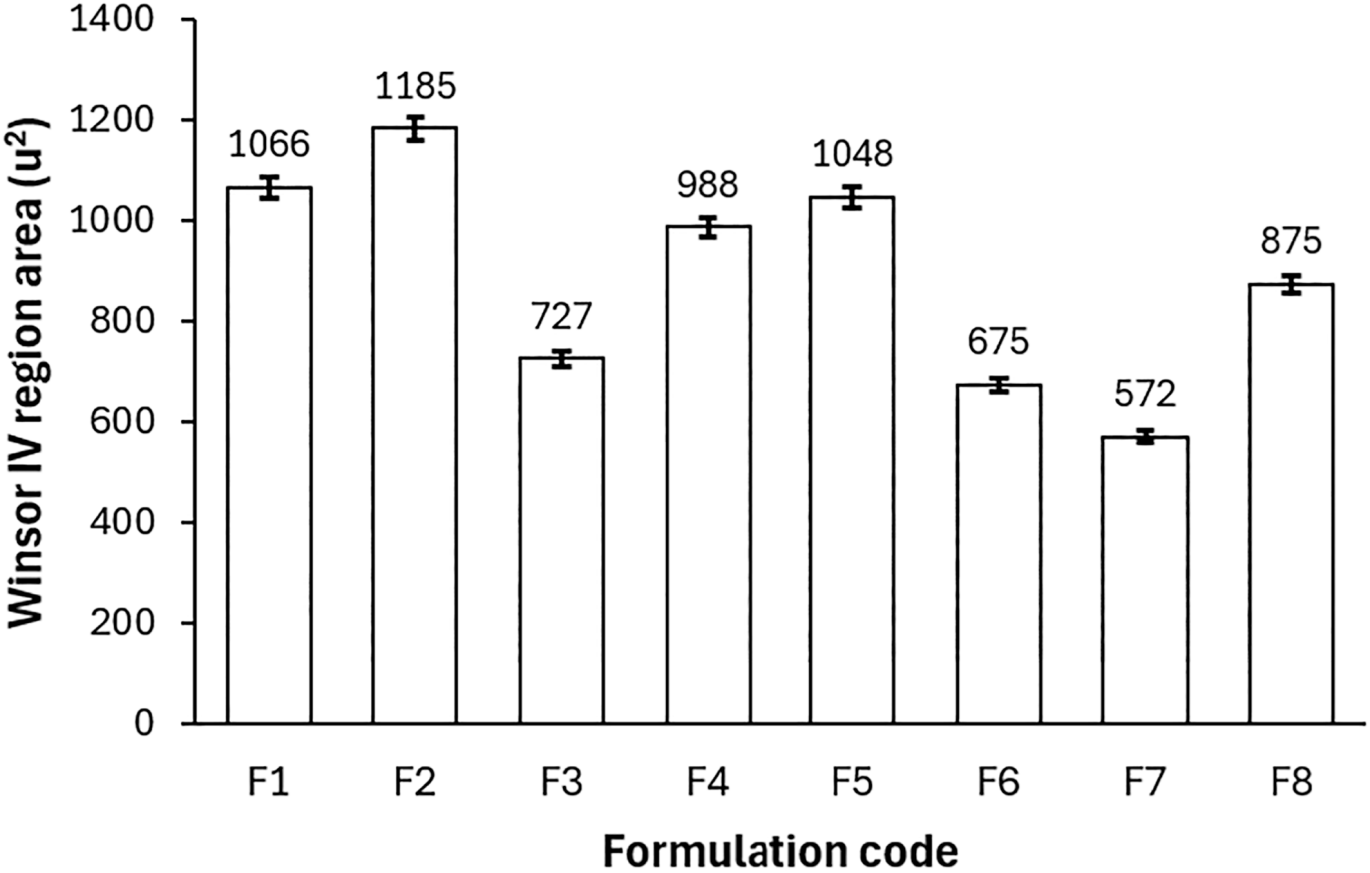
Comparison of the single-phase (Winsor IV) region areas for formulations F1–F8, calculated via numerical integration from pseudoternary phase diagrams (C/S ratio = 10). Areas in normalized arbitrary units (u^2^) reflect thermodynamic stability robustness, with larger values indicating higher tolerance to compositional variations. Data represent means of triplicate (standard deviation < 2%).

As shown in Figure 2, formulation F2 achieved the largest single-phase region (1185 ± 24 u^2^, encompassing 27.4% of the total diagram area), signifying exceptional thermodynamic stability. This biobased system, combining pine oil, saponified coconut oil, ethyl alcohol, and distilled water, outperforms its analogue F1 (1066 ± 21 u^2^, using isopropyl alcohol) by leveraging the shorter carbon chain of ethanol (C2 vs C3), which enhances interfacial packing and flexibility, a novel insight into cosurfactant optimization for biobased Winsor IV microemulsions. In stark contrast, F7 (with 2% NaCl) displayed the smallest area (572 ± 11 u^2^, 13.2% of total), attributable to the ionic strength sensitivity of the anionic saponified coconut oil; elevated salinity compresses the electrical double layer,^35,36^ diminishing anionic group repulsion and shrinking the stability region.^37^ This finding innovatively demonstrates how biobased surfactants can maintain Winsor IV integrity under moderate salinity, a resilience not commonly reported for renewable systems.

The surfactant-nonpolar phase interaction emerged as a pivotal “puzzle piece” in driving novelty. Formulations with pine oil showed comparable areas between biobased and conventional surfactants (F1: 1066 ± 21 u^2^ with saponified coconut oil vs F5: 1048 ± 21 u^2^ with Triton X-100), indicating broad compatibility. However, with toluene, the biobased surfactant excelled (F4: 988 ± 20 u^2^ vs F8: 875 ± 17 u^2^), owing to electrostatic repulsion forming a more adaptable interfacial film, unlike the rigid, sterically driven interface of Triton X-100. The toluene–Triton X-100 pairing (F8) yielded the least synergistic nonsaline system, underscoring the limitations of conventional aromatics.

These outcomes build on but advance prior work with functionalized natural oils like pine oil^32,38^ and d-limonene,^20,21,39–41^ by pioneering their application in comparative Winsor IV contexts. Replacing toxic toluene with biodegradable pine oil (rich in terpenes) and Triton X-100 with renewable saponified coconut oil not only mitigates environmental impact but also amplifies phase stability,^2,42^ an approach to sustainable EOR with superior adaptability for field deployment. dual benefit is absent in most literature. The interplay between these expanded Winsor IV regions and key properties like IFT and viscosity, detailed in Section 3.2, further emphasizes the synergistic efficiency of our biobased “puzzle” assembly, offering a transformative.

### 3.2 Physicochemical Characterization of Winsor IV Microemulsions

Dynamic viscosity is a critical hydrodynamic parameter in evaluating fluid displacement performance.^43,44^ A central finding of this comparative study is that most formulations, regardless of whether their constituents are synthetic (F1, F2, F4) or saline-based (F6, F7), exhibit remarkably low viscosities ranging from 1.1 to 1.4 cP at 60 °C.^8^ These values closely approximate that of injection water, which enhances injectivity and reduces the risk of rock fracturing, thereby enabling application in low-permeability reservoirs.^19,45^ However, this favorable low viscosity introduces a significant limitation in the form of a high mobility ratio (*M* > 1)^2,43^ which typically leads to viscous fingering and poor sweep efficiency, persistent challenges in chemical EOR operations.^5,46^

It is in this context that the biobased formulation strategy exhibits its most compelling innovation. In formulation F3, the strategic replacement of distilled water with glycerol, a renewable byproduct of biodiesel production, results in a 4-fold increase in viscosity to 4.13 ± 0.03 cP (Figure 3) while maintaining a stable Winsor IV phase behavior. This outcome represents more than a conventional solvent substitution; it reflects a structural advancement in formulation design.

**Figure 3:**
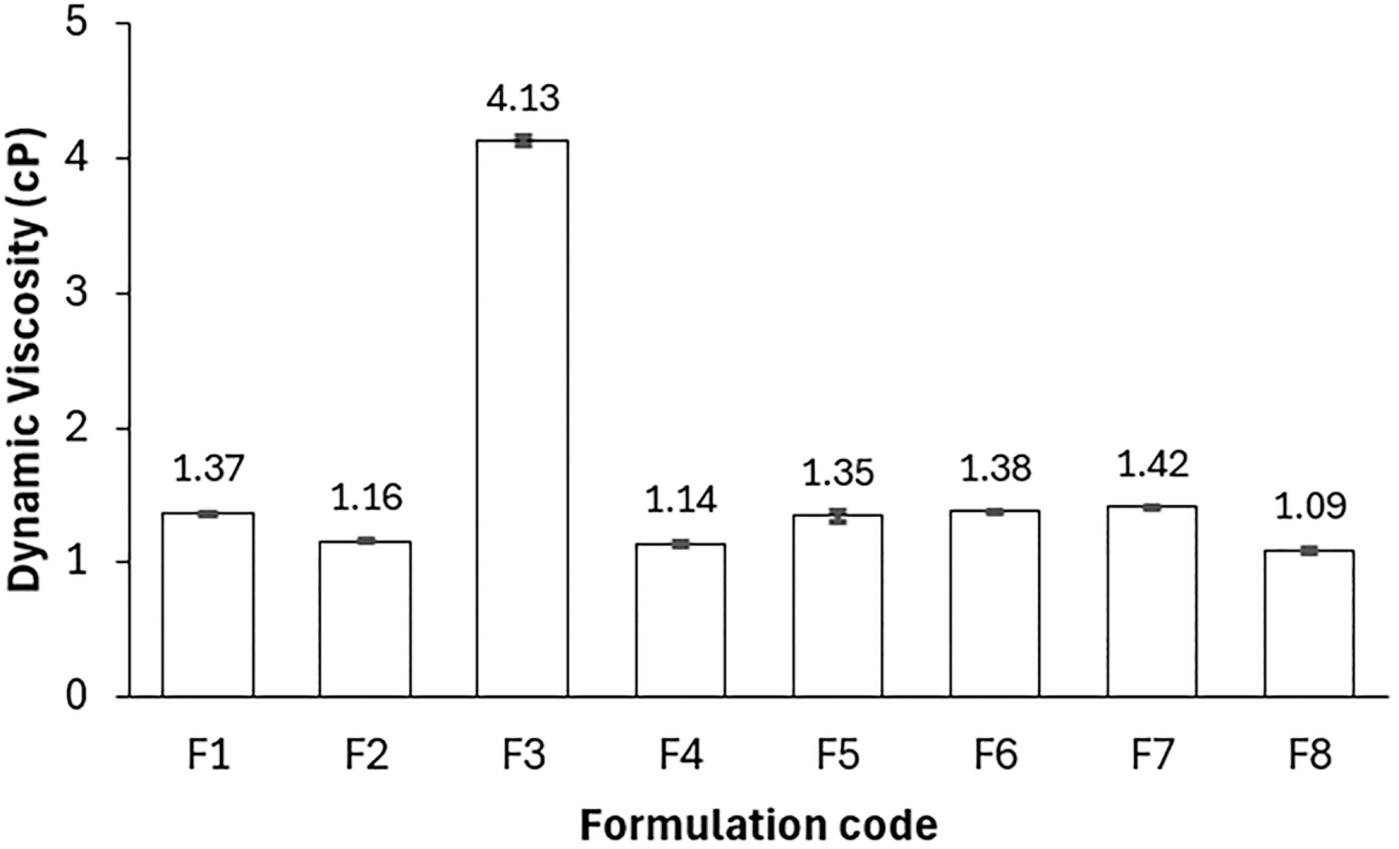
Dynamic viscosity of microemulsions F1–F8 at 60 °C, highlighting the influence of glycerol on the rheological behavior.

Unlike traditional ASP systems that rely on the addition of polymers for mobility control, glycerol in F3 functions as an integral structural component of the microemulsion. It modulates the polarity of the aqueous phase while elevating the viscosity into the target operational window (3–9 cP), thus achieving co-optimization of interfacial and hydrodynamic properties within a single-phase system. Furthermore, the F3 formulation, composed exclusively of coconut oil-derived surfactant, pine oil, and glycerol, addresses the dual challenge of injectivity and mobility control through a fully biobased composition without the need for polymers or high salinity.

Consequently, F3 emerges not merely as a viable alternative but as a paradigm shift in EOR formulation design. It exemplifies how the intelligent integration of renewable components can resolve the injectivity–mobility paradox in a thermodynamically stable, single-phase system, offering both environmental and operational advantages for next-generation oil recovery strategies.

Building on the expanded Winsor IV regions in biobased formulations (Section 3.1), our comparative analysis reveals physicochemical synergies that propel these systems beyond conventional benchmarks, particularly in achieving low IFT with tunable viscosities under reservoir-representative conditions (∼60 °C). This novelty lies in harnessing pine oil’s terpenoid functionality and saponified coconut oil’s anionic charge to forge adaptive interfaces (interfaces capable of modulating their curvature and molecular organization in response to changes in the local environment), enabling Winsor IV stability in saline and glycerol environments, regimes where prior biobased efforts faltered toward Winsor I/III phases.^32–34^ Such integrations not only mitigate environmental toxicity but also yield formulations with superior injectability and oil mobilization potential, addressing a pivotal gap in scalable, green EOR chemistries.^2,3^

Specific densities ranged from 0.8442 to 0.8946 g·cm^–3^ (Figure 4A), with F3′s elevated value (0.8946 ± 0.0005 g·cm^–3^) underscoring glycerol’s role in densifying the polar phase without disrupting Winsor IV translucency, a strategic enhancement for mobility control in heterogeneous reservoirs, diverging from lower densities in analogous systems.^1^ Concurrently, refractive indices (1.351–1.393; Figure 4B) signal compact microstructures in polar-enriched formulations, amplifying interfacial cohesion in biobased variants.

**Figure 4:**
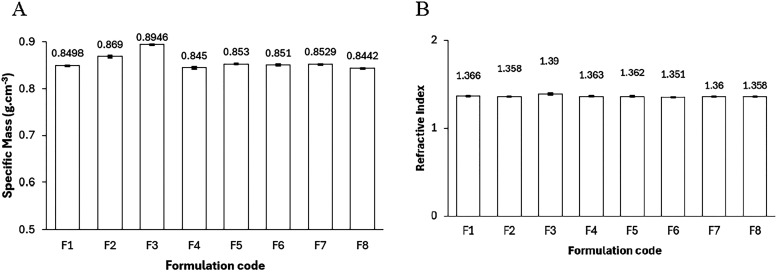
(A) Specific density (g·cm^–3^) and (B) refractive index variations across F1–F8. Glycerol in F3 drives peak density, fostering cohesive polar phases that bolster thermodynamic stability in biobased microemulsions.

Electrical conductivities (Figure 5A) delineate structural morphologies: Triton X-100 systems (F5: 110 ± 8 μS·cm^–1^; F8: 77 ± 5 μS·cm^–1^) imply minimal aqueous percolation, suggestive of O/W-inverted domains akin to prior low-conductivity reports,^1,2^ whereas saponified coconut oil’s formulations (F1, F2, F3, F4, F6, F7: 2800–4223 μS·cm^–1^) affirm aqueous-continuous phases, aligning with ionic surfactant paradigms.^4^ pH values (Figure 5B) reinforce this dichotomy, with alkaline profiles (∼9–10) in biobased systems enhancing charge-driven stability, versus near-neutrality in nonionics.

**Figure 5:**
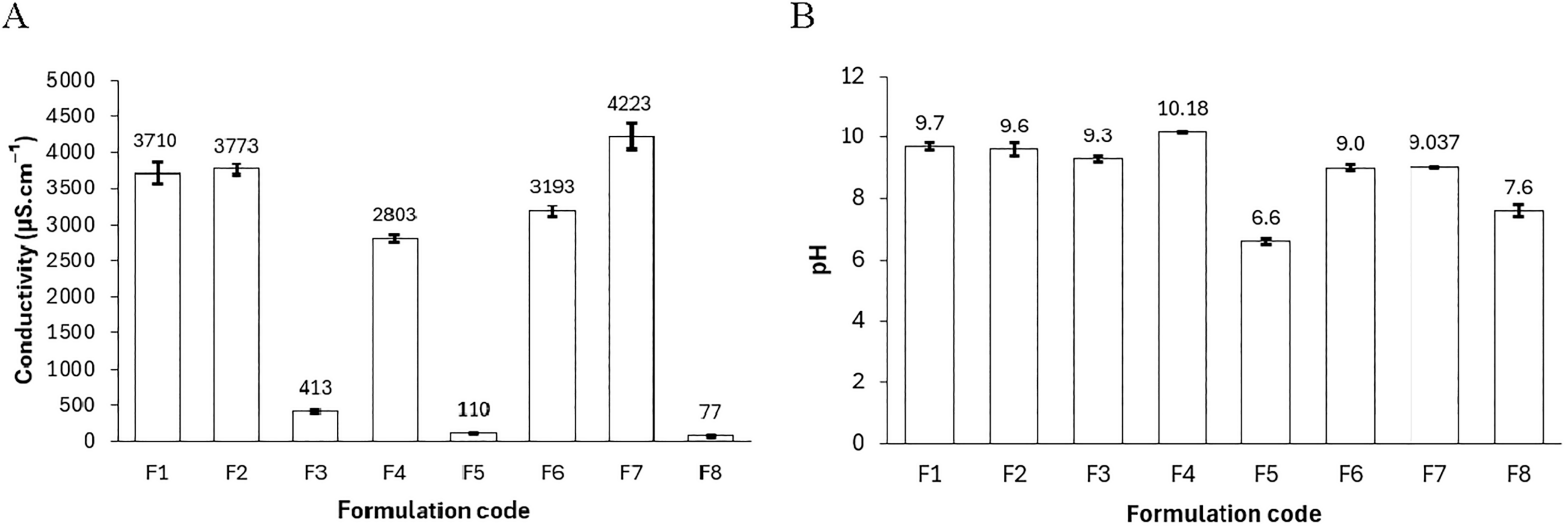
(A) Electrical conductivity and (B) pH of microemulsions F1–F8 at 60 °C. Systems with Triton X-100 (F5, F8) showed low conductivities (110–77 μS·cm^–1^), indicating a W/O structure, while those with saponified coconut oil (F1, F2, F4, F6, F7) exhibited higher values (2800–4223 μS·cm^–1^), consistent with an aqueous-continuous phase. The pH remained alkaline for saponified coconut oil systems and near neutral for those with Triton X-100.

Surface tensions consistently decreased to the range of 22–25 mN·m^–1^ across all formulations (Figure 6A), representing a substantial reduction from the baseline value of pure water (72 mN·m^–1^) and confirming the interfacial activity of the employed surfactants.^3^ However, it is the IFT that underscores the most significant advancement in this study. Biobased systems (F1–F3, F6, F7) demonstrated IFTs ranging from 2.7 ± 0.1 to as low as 0.14 ± 0.01 mN·m^–1^, with formulation F7 achieving the minimum under 2% NaCl (Figure 6B). This low IFT approaches the optimal salinity window described in the literature^7^ and highlights the capacity of bioderived interfaces to maintain functional integrity under saline conditions relevant to EOR applications.

**Figure 6:**
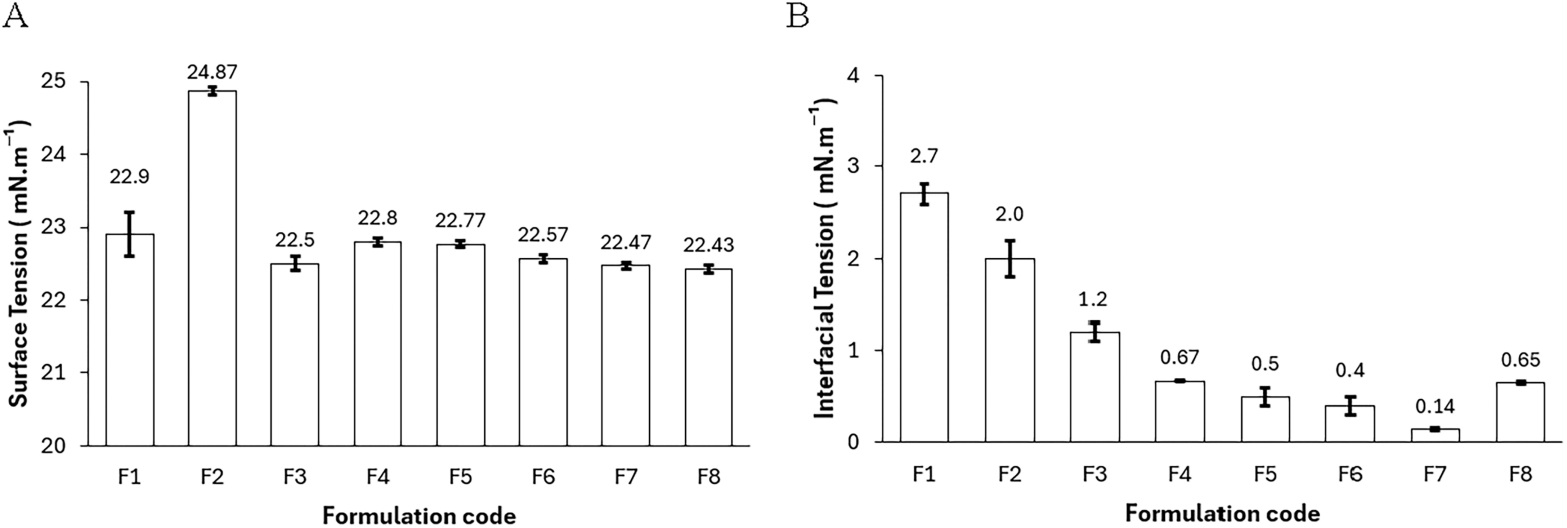
(A) Surface tension values (air/microemulsion) measured at 60 °C. All formulations effectively reduced the surface tension to the range of 22–25 mN·m^–1^. (B) Interfacial tension values (oil/microemulsion) measured at 60 °C. Formulation F7 (2% NaCl) exhibited the lowest value (0.14 mN·m^–1^), indicating high interfacial efficiency.

An inverse trend emerged between Winsor IV extension and IFT: formulations with broader monophasic regions generally exhibited lower IFT, although the relationship was not strictly linear. F7 illustrates this nonlinearity, as its very low IFT (0.14 ± 0.01 mN·m^–1^) coincides with salinity-restricted stability. In contrast, F5 combines low IFT (0.5 ± 0.1 mN·m^–1^) with a broad Winsor IV region due to the pine oil–Triton synergy. On the other hand, F8 shows a higher IFT (0.65 ± 0.01 mN·m^–1^) and a narrow Winsor IV region, reflecting the mismatch between aromatic oils and nonionic surfactants.^3^ The salinity-dependent trend (F1 > F6 > F7) further supports ionic-interaction optima.^7^

Practically, viscosities ≤ 4 cP paired with IFT < 0.5 mN·m^–1^ in F5, F6, and F7 optimize capillary desaturation and injectability, minimizing viscous fingering in porous media,^9,21^ a biodriven advancement for enhanced sweep efficiency. These findings coalesce within the formulation “puzzle” framework, wherein each constituent plays a synergistic role in directing interfacial architecture. Surfactant–cosurfactant pairs govern film flexibility, with biobased anionic surfactants facilitating the formation of elastic interfacial layers.^19,39^ Nonpolar components such as pine oil, enriched with oxygenated moieties, promote dynamic self-assembly,^17^ surpassing the interfacial rigidity imposed by aromatic hydrocarbons like toluene.

Modulation of the polar phase through salinity or glycerol incorporation enables precise curvature tuning, thereby expanding the thermodynamically stable Winsor IV domain.^32^ When component ratios are optimally configured, the system achieves minimal interfacial tension alongside maximal stability;^16,29^ conversely, compositional mismatches trigger phase destabilization. This cooperative orchestration of bioderived elements provides a strategic blueprint for designing next-generation microemulsions, enabling exceptional interfacial performance and environmental compatibility for advanced oil recovery applications.

Oil recovery by microemulsion slug injection arises from the integrated action of physicochemical mechanisms that modify the balance between capillary, viscous, and adhesive forces responsible for residual oil trapping in porous media.^47^ The substantial reduction in oil–water interfacial tension increases the capillary number to levels that enable the deformation and mobilization of oil ganglia through pore throats that are otherwise inaccessible to flow.^47,48^ In parallel, adsorption of surfactants from the microemulsion onto the rock surface promotes the displacement of polar oil components, inducing wettability alteration toward more water-wet conditions and weakening oil–rock adhesive interactions.^27,48^

During slug propagation, the interaction between the microemulsion and residual oil favors in situ emulsification and partial solubilization of oil into the displacing phase. The transient formation of emulsified droplets may generate temporary local flow restrictions in high-permeability channels, redirecting flow toward less-swept regions of the porous medium.^47^ Additionally, the higher viscosity and favorable rheological behavior of microemulsions contribute to mobility control by suppressing pore-scale flow instabilities.^27,48^ Collectively, these mechanisms provide a consistent physicochemical framework for interpreting residual oil mobilization during microemulsion slug injection, in agreement with mechanistic descriptions reported in classical and recent literature.^47^

To place the physicochemical results presented above in perspective, a quantitative benchmarking was performed comparing the biobased microemulsion formulations (F1, F2, F3, and F7) with both conventional petrochemical systems reported in the literature and the petrochemical reference formulation developed in this study (F8). The most significant advancement concerns thermodynamic phase stability. The biobased formulation F2 exhibited a single-phase Winsor IV area of 1185 u^2^, corresponding to a 35.4% increase relative to the petrochemical reference system F8 (875 u^2^). In contrast, conventional microemulsions based on synthetic surfactants such as sodium dodecyl sulfate typically operate within narrow salinity windows and are prone to phase transitions or precipitation under compositional perturbations.^21,24^ The expanded Winsor IV domain observed for pine-oil-based systems, therefore, reflects enhanced compositional resilience under dilution and salinity variations expected in reservoir environments.

Regarding interfacial performance, optimized synthetic systems based on methyl ester sulfonates or SDS can achieve ultralow interfacial tensions on the order of 10^–3^ mN·m^–1^.^16,24^ By comparison, formulation F7 (2% w/v NaCl) achieved an interfacial tension of 0.14 ± 0.01 mN·m^–1^, which, although higher, remains within the low-IFT regime considered sufficient for residual oil mobilization when combined with favorable phase stability and mobility control. Consistently, biodiesel-derived and natural surfactant systems reported in the literature have achieved tertiary oil recoveries of approximately 39–40% at comparable IFT levels, indicating that recovery efficiency depends on the synergy between interfacial tension reduction and sweep efficiency rather than on ultralow IFT alone.^19^

In addition, formulation F3, containing glycerol, reached a viscosity of 4.13 cP, substantially higher than that of water (0.47 cP at 60 °C) and conventional low-viscosity microemulsions. Unlike ASP strategies that require polymer addition to achieve mobility control,^8,49^ the F3 system provides intrinsic viscosity enhancement within a single-phase microemulsion. This behavior is consistent with previous reports showing that glycerol-based microemulsions improve sweep efficiency without polymer incorporation.^32^

Overall, this quantitative benchmarking demonstrates that the biobased formulations proposed herein outperform the petrochemical reference system in thermodynamic stability while offering a balanced combination of interfacial tension reduction and mobility control. These performance gains are achieved using renewable components, reducing reliance on aromatic solvents such as toluene, and reinforcing the technical competitiveness of biobased microemulsions for chemical enhanced oil recovery applications.^28^

### 3.3 Influence of the Nonpolar Phase on Winsor IV Microemulsion Stability

Our investigation unveils a groundbreaking disparity in Winsor IV region extents driven by nonpolar phase selection, with pine oil-based formulations (F1–F3, F5–F7) yielding up to 35% larger monophasic areas than toluene counterparts (F4, F8), a novel outcome attributing to pine oil’s terpenoid and oxygenated moieties fostering unprecedented interfacial adaptability in biobased EOR systems. This advancement challenges conventional reliance on aromatic hydrocarbons, demonstrating how renewable, functionalized nonpolars like pine oil enable resilient microemulsions under variable salinity and temperature, a synergy underexplored in the prior Winsor IV literature.^16,20^

Pseudoternary phase diagrams (Figures 7 and 8) contrast pine oil (F1: pine oil, saponified coconut oil, isopropyl alcohol, distilled water) and toluene (F4: analogous but with toluene) systems, revealing pine oil’s cooperative interactions with surfactant films that expand Winsor IV domains and suppress interfacial tensions. Toluene’s purely nonpolar aromaticity, conversely, imposes rigidity, necessitating elevated surfactant loads for comparable stability, a limitation echoed but amplified here through direct bioconventional benchmarking.^20^

**Figure 7:**
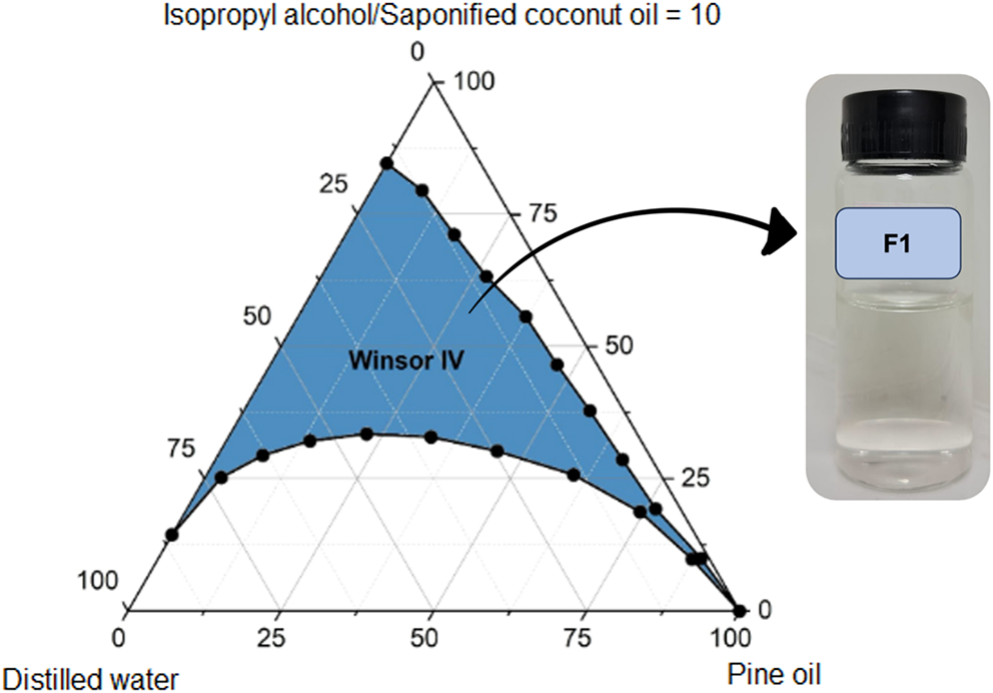
Pseudoternary phase diagram of formulation F1 (pine oil, saponified coconut oil, isopropyl alcohol, distilled water), showing an expanded Winsor IV region within the biobased formulation set, associated with favorable interfacial synergy among plant-derived components.

**Figure 8:**
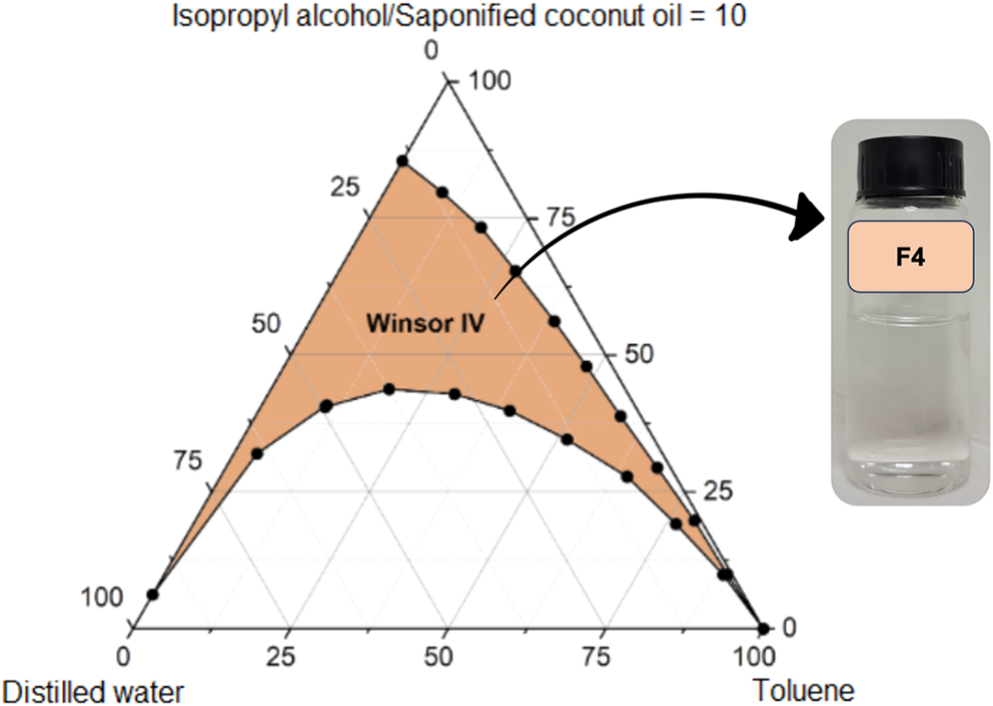
Pseudoternary phase diagram of formulation F4 (toluene, saponified coconut oil, isopropyl alcohol, distilled water), displaying a more restricted Winsor IV region when compared with the corresponding biobased formulation, indicating reduced interfacial adaptability of the petrochemical oil phase.

The selection of toluene and pine oil as model oil phases in this study is therefore grounded in the need to rationalize the physicochemical mechanisms underlying the differences observed in Winsor IV stability, while avoiding the confounding effects associated with the highly variable composition of crude oil. Unlike linear alkanes, aromatic and terpenoid solvents exhibit a stronger affinity for polar crude oil fractions such as resins and asphaltenes, owing to more compatible Hansen solubility parameters.^17^ In particular, pine oil has been associated with viscosity reduction and mitigation of asphaltene precipitation, thereby contributing to the preservation of single-phase integrity upon contact with residual oil in the reservoir.^17,32,40^ Accordingly, the transferability of results obtained with model oil phases to real reservoir systems is supported by the concepts of Equivalent Alkane Carbon Number (EACN) and Hydrophilic–Lipophilic Deviation (HLD), according to which crude oil can be treated as a pseudocomponent exhibiting equivalent interfacial behavior when the model oil phase is tuned to a compatible EACN.^50–52^ Moreover, the intrinsic polarity of these oil phases promotes the formation of broader and more stable Winsor IV regions, positively impacting rheological behavior and mobility control in heavy oil reservoirs.^5,9,32^

Extending beyond Qin et al.^20^ and Pal et al.,^16^ who noted enhanced solubilization with polar-moderate oils in isolated contexts, our work pioneers pine oil’s “molecular flexibility” (defined as the ability of amphiphilic molecules to undergo conformational adjustments at the interface to optimize molecular packing and promote interfacial stability) in comparative Winsor IV frameworks, where terpene-oxygenated architectures dynamically interface with cosurfactants, yielding robust monophasicity without the chemical overhead of hydrocarbon systems.^35,36,40^

For EOR implementation, these findings indicate that biofunctionalized nonpolar phases, such as pine oil, provide enhanced compositional resilience, maintaining interfacial integrity and mobilization efficiency across variable reservoir conditions. Unlike toluene-based systems, which demand precise surfactant–cosurfactant tuning to preserve monophasicity, pine oil formulations exhibit broader stability windows with reduced chemical demand. This not only simplifies field-scale formulation strategies but also aligns with regulatory and operational goals for reduced ecotoxicity and reliance on petrochemical-derived inputs.

### 3.4 Effects of Polar Phase Composition on Microemulsion Stability and Mobility Control

In microemulsion formulation, the polar phase is not a passive solvent matrix, it operates as a pivotal, tunable component that defines interfacial architecture, rheological behavior, and thermodynamic robustness. This section presents a strategically comparative analysis of two fundamentally distinct approaches to polar phase design within a renewable Winsor IV matrix (pine oil/saponified coconut oil/isopropyl alcohol): a salinity-driven route, conventionally adopted in EOR to modulate hydrophilic–lipophilic balance (HLB) and interfacial tension (IFT), and a polyol-based route, leveraging glycerol to modulate viscosity and polarity within a structurally coherent, biobased formulation.

The incorporation of NaCl at 1% w/v (F6) and 2% w/v (F7), Figures 9 and 10, respectively, leads to a pronounced contraction of the Winsor IV domain, with F7 exhibiting the lowest phase stability in the study (572 ± 11 u^2^). This reflects classic ionic strength effects: compression of the electrical double layer surrounding anionic headgroups, reduced electrostatic repulsion, and a subsequent loss in interfacial flexibility. While such effects are well documented in the literature, their destabilizing impact within fully biobased Winsor IV systems, as shown here for the first time, raises critical formulation concerns. Unlike conventional EOR systems that aim to optimize salinity to induce Winsor III behavior, our data (Figures 9 and 10) indicate that salinity adjustments are counterproductive for sustaining single-phase stability in renewable Winsor IV formulations. These findings challenge long-held assumptions about universal salinity optimization and highlight the formulation-specific trade-offs that emerge when adopting bioderived surfactants.

**Figure 9:**
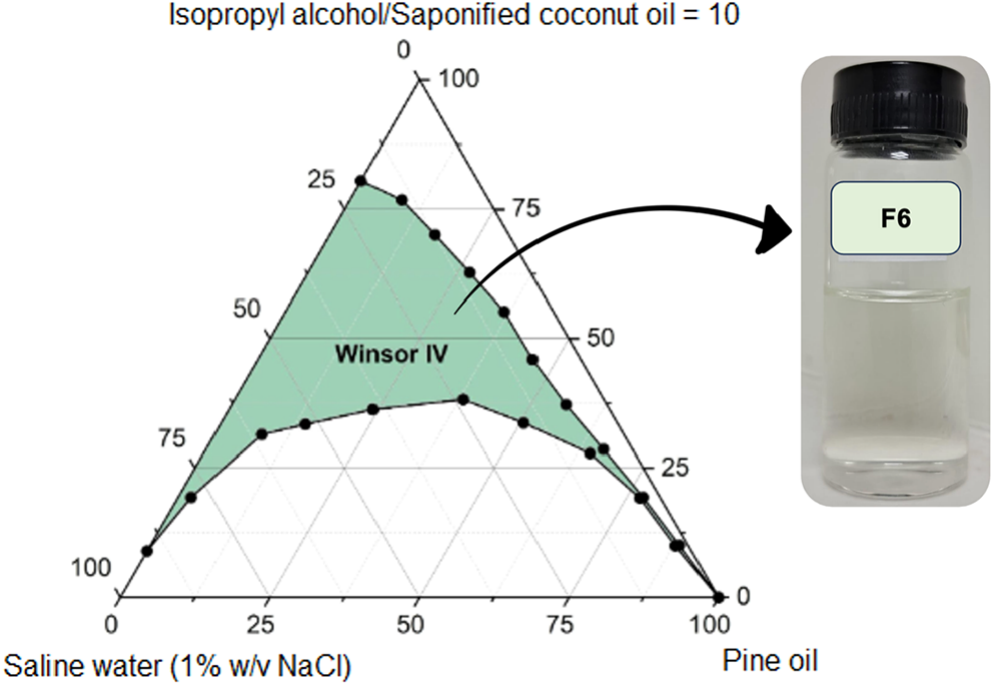
Pseudoternary phase diagram of formulation F6 (pine oil, saponified coconut oil, isopropyl alcohol, saline water at 1% w/v NaCl), illustrating the contraction of the Winsor IV region induced by moderate salinity within the biobased system.

**Figure 10:**
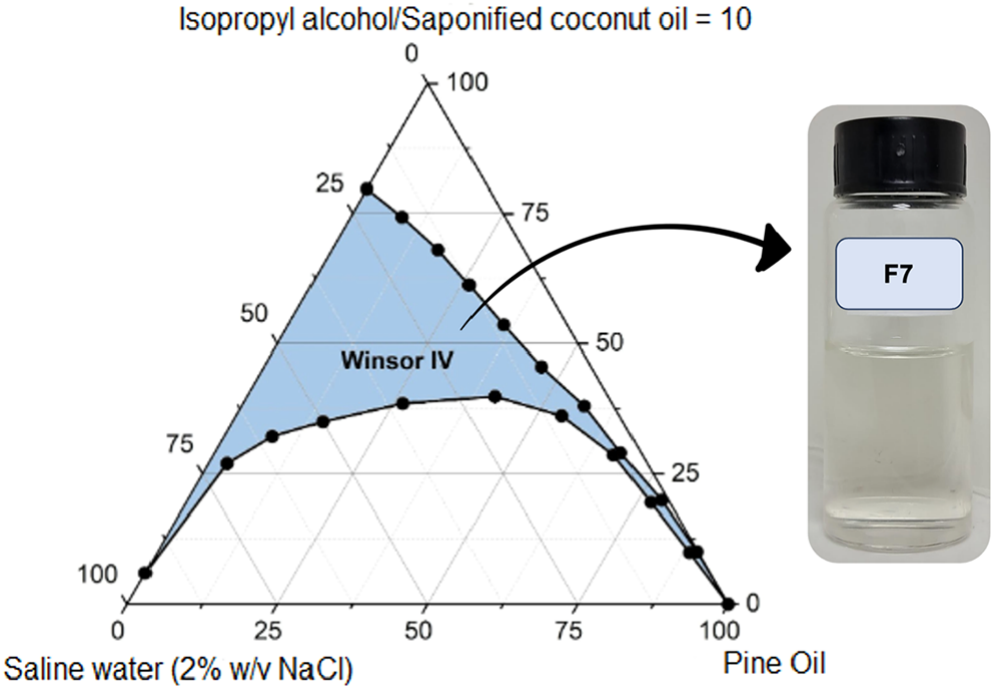
Pseudoternary phase diagram of formulation F7 (pine oil, saponified coconut oil, isopropyl alcohol, saline water at 2% w/v NaCl), showing a further reduction of the Winsor IV region as salinity increases, reflecting salinity-induced limitations on interfacial film flexibility.

In stark contrast, the use of glycerol (F3) as a polar phase modifier maintains a broad and robust Winsor IV region (1048 ± 21 u^2^, Figure 11), while simultaneously enhancing viscosity (4.13 ± 0.03 cP) and density (0.8946 ± 0.0005 g·cm^–3^). This dual-function behavior arises from glycerol’s hydrogen-bonding capabilities, which increase molecular cohesion and interface strength, resulting in superior resistance to coalescence and improved phase integrity under reservoir conditions. Crucially, the elevated viscosity aligns with an optimal mobility ratio (*M* < 1), mitigating the fingering and displacement inefficiencies often associated with low-viscosity aqueous systems. Unlike prior studies where glycerol served only as a cosolvent, here it functions as a core formulation element, enabling polymer-free mobility control in a thermodynamically stable, biobased system.

**Figure 11:**
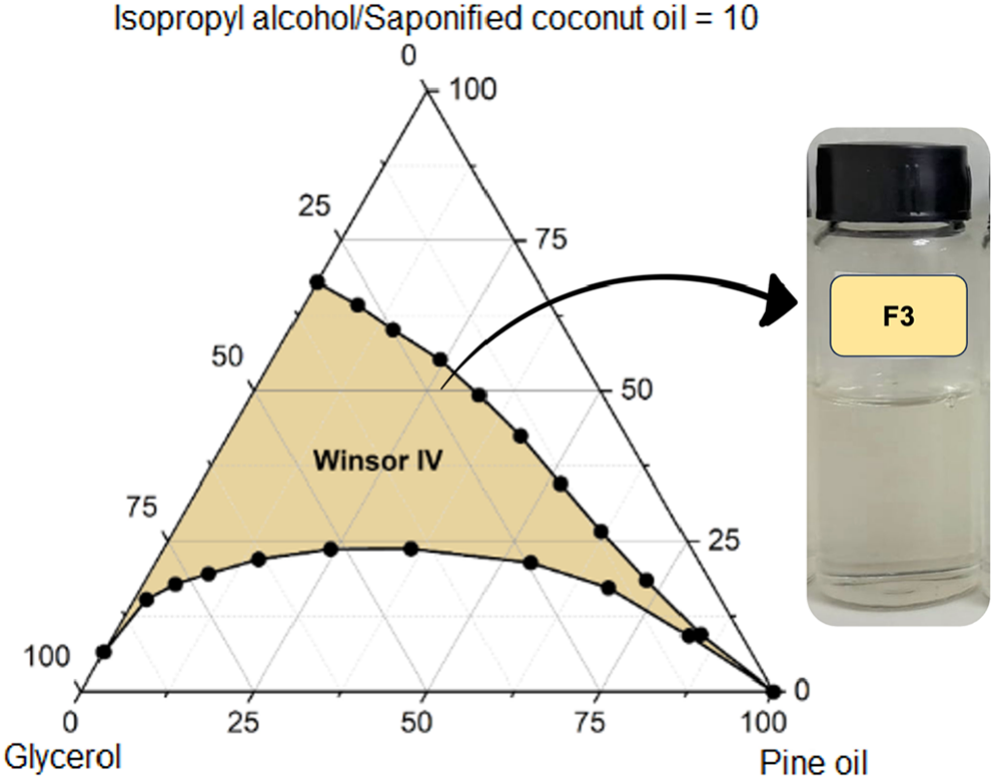
Pseudoternary phase diagram of formulation F3 (pine oil, saponified coconut oil, isopropyl alcohol, glycerol), exhibiting an intermediate Winsor IV region within the biobased formulations, reflecting the influence of glycerol on phase behavior and interfacial organization.

Our comparative analysis provides a compelling design directive: while saline routes compromise stability in biobased Winsor IV systems, glycerol offers a structurally integrated and environmentally benign alternative that solves the injectivity–mobility paradox without chemical complexity. The F3 formulation exemplifies this innovation, functioning as a self-sufficient EOR slug that unifies thermodynamic resilience, interfacial performance, and flow control in a monophase architecture.

While the comparative results indicate superior interfacial efficiency and phase robustness of selected biobased formulations relative to petrochemical analogues, this performance must be interpreted within the investigated operational window. The formulations evaluated herein remained structurally stable and maintained ultralow interfacial tension at 60 °C and under the salinity conditions tested, indicating adequate resistance to thermal and ionic stresses relevant to many sandstone reservoirs. However, unlike highly fluorinated or sulfonated synthetic surfactants, biobased systems may be susceptible to degradation pathways such as ester hydrolysis or oxidative reactions under prolonged exposure to extreme temperatures or highly alkaline environments. Literature reports indicate that these limitations can be mitigated through formulation strategies, including surfactant blending, cosurfactant optimization, and the incorporation of stabilizing agents or nanoparticles, which enhance salinity tolerance and delay chemical degradation.^16,19,24^

From a long-term perspective, the operational window discussed above must be interpreted in light of phase behavior evolution under extended thermal exposure and salinity stress. Recent aging studies demonstrate that eco-friendly microemulsion systems formulated with biobased surfactants and renewable oil phases are capable of preserving micellar integrity and macroscopic homogeneity over prolonged periods, even under combined thermal and saline conditions. In particular, Santos et al.^17^ reported that green microemulsion-based formulations maintained phase stability, transparency, and functional performance during long-term exposure to elevated temperatures and saline environments, with gradual and predictable phase behavior evolution rather than abrupt phase separation. These findings indicate that flexible, bioderived interfacial films can reorganize under physicochemical stress while preserving single-phase integrity.

In this context, the sustained Winsor IV behavior and low interfacial tensions observed in the present study at 60 °C and under saline conditions suggest that the proposed formulations possess the thermodynamic and kinetic attributes required to tolerate extended residence times in porous media. Although dedicated long-term aging experiments were beyond the scope of this work, the expanded Winsor IV domains, combined with insights from recent aging studies, support the interpretation that the investigated biobased systems offer a robust operational window for EOR applications.

In conclusion, the polar phase is not merely a diluent; it is a precision-tuned variable whose composition dictates interfacial curvature, dynamic behavior, and long-term reservoir performance. Mastering its role within the formulation “puzzle” is essential for advancing next-generation, biobased EOR microemulsions with field-ready applicability and minimal environmental burden.

### 3.5 Surfactant–Cosurfactant Interactions in Winsor IV Systems

The cooperative interaction between surfactant and cosurfactant emerged as a key determinant in shaping the extent and stability of the Winsor IV regions in the studied microemulsions. Formulations composed of saponified coconut oil combined with short-chain alcohols such as ethanol or isopropanol (F1–F3, F4, F6, F7) consistently exhibited broader monophasic domains than those incorporating the nonionic surfactant Triton X-100 (F5, F8), as shown in Figures 12–14, where formulations F5, F8, and F2 reveal the most distinct variations in their Winsor IV domains. This contrast underscores the critical role of surfactant charge and interfacial packing flexibility in promoting thermodynamic stability.

**Figure 12:**
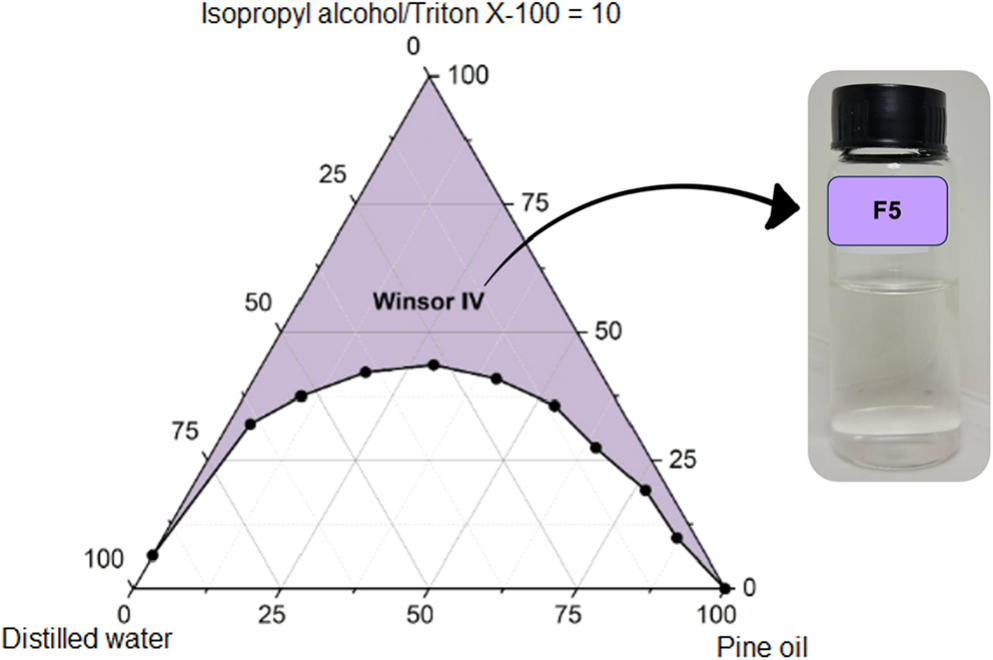
Pseudoternary phase diagram of formulation F5 (pine oil, Triton X-100, isopropyl alcohol, distilled water), showing the most expanded Winsor IV region within the petrochemical surfactant formulation set, highlighting the effectiveness of Triton X-100 under the investigated conditions.

**13:**
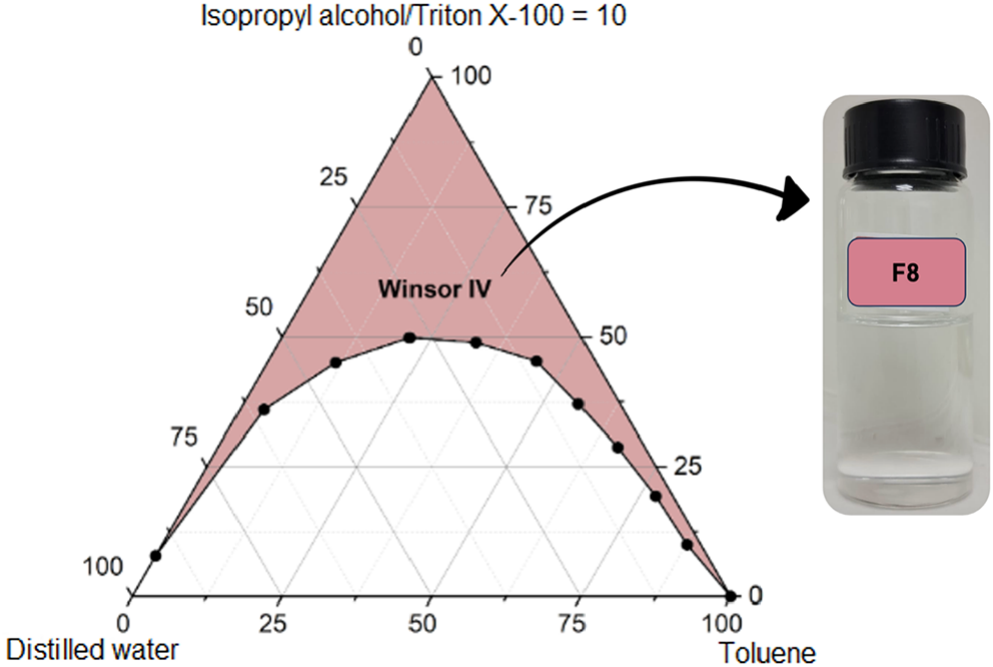
Pseudoternary phase diagram of formulation F8 (toluene, Triton X-100, isopropyl alcohol, distilled water), presenting a reduced Winsor IV region relative to F5, evidencing the influence of the nonpolar phase on interfacial organization in petrochemical systems.

**Figure 14:**
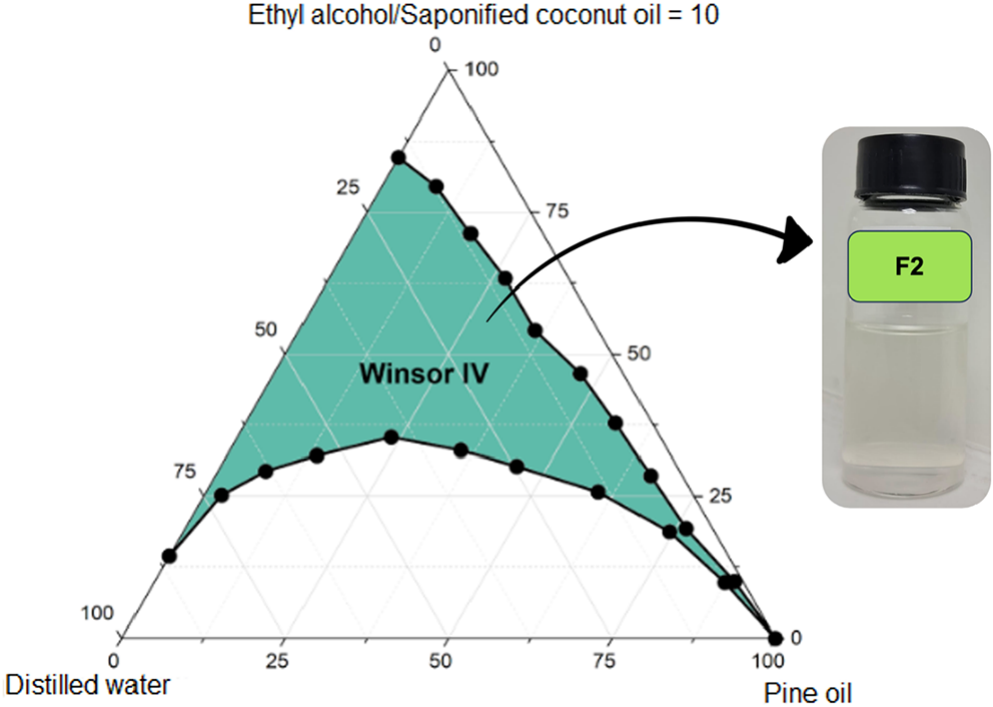
Pseudoternary phase diagram of formulation F2 (pine oil, saponified coconut oil, ethyl alcohol, distilled water), demonstrating a more pronounced expansion of the Winsor IV region within the biobased formulation series when compared with isopropyl alcohol, attributed to the smaller molecular size of ethanol and its improved interfacial packing within the surfactant film.

Pseudoternary phase diagrams reveal that anionic, bioderived surfactant–cosurfactant pairs generate interfacial films that are both more resilient and more elastic than those formed by their petrochemical counterparts. While the Triton-based formulation F5 achieved a considerable monophasic area (1048 ± 21 u^2^), its biobased analogue F2 surpassed this value (1185 ± 24 u^2^), confirming that the presence of ionic headgroups and hydrogen-bonding potential enhances interfacial adaptability and structural tolerance.

In contrast, systems based on Triton X-100 (F5, F8) achieved low interfacial tensions (0.5–0.65 mN·m^–1^) yet displayed narrower Winsor IV regions. Notably, formulation F8 (Triton X-100 with toluene) presented the smallest monophasic domain among the nonsaline systems (875 ± 17 u^2^), indicating that low interfacial tension alone does not guarantee thermodynamic robustness. This finding aligns with prior reports by Aum et al.^40^ and Qin et al.,^20^ which emphasize that interfacial flexibility is equally critical for the persistence of stable microemulsions in dynamic reservoir conditions.

A key mechanistic insight arises from the comparison between cosurfactants. Ethanol, with its smaller molecular size and higher polarity, promoted a more efficient interfacial fit than isopropanol. This was evidenced by the broader Winsor IV region in formulation F2 (Figure 14). The compact structure of ethanol likely allows for deeper penetration into the surfactant layer, reducing interfacial rigidity and optimizing the HLB, thereby enhancing the stability and curvature adaptability of the microemulsion.

Altogether, these findings redefine the role of the surfactant–cosurfactant pair as a dynamic, interdependent design element within the formulation “puzzle”. Anionic natural surfactants combined with short-chain alcohols produce interfacial networks that are simultaneously flexible, compositionally tolerant, and capable of maintaining low interfacial tensions under the complex gradients of temperature and salinity typical of EOR operations.

The novelty of this work lies in demonstrating that bioderived surfactant–cosurfactant pairs not only match but can outperform conventional systems in both interfacial performance and adaptability. Their structural versatility, cooperative interfacial dynamics, and enhanced phase resilience make them strong candidates for sustainable, high-performance EOR formulations.

### 3.6 Implications for Enhanced Oil Recovery (EOR)

In general, the “puzzle” analogy emphasizes that optimal EOR performance is not achieved through isolated properties but by harmonizing molecular interactions throughout the entire formulation. The results demonstrate that the performance of the biobased microemulsions developed in this study is directly linked to the precise tuning of interactions among the surfactant, cosurfactant, and the polar and nonpolar phases. This molecular arrangement promotes the formation of thermodynamically stable systems characterized by ultralow interfacial tensions and extensive Winsor IV regions, which are essential for effective EOR applications.^29,40^

The main novelty of this work lies in the use of naturally derived components, particularly saponified coconut oil and functionalized natural oils such as pine oil, which exhibited interfacial synergy comparable to or even superior to that of conventional petrochemical systems. This biobased combination resulted in more flexible and adaptive amphiphilic films capable of reorganizing in response to variations in salinity and the composition of the nonpolar phase, while maintaining stability and low interfacial tension. Previous studies have indicated that systems with high molecular mobility, such as those incorporating short- or medium-chain alcohols, tend to produce more flexible and efficient interfaces for oil displacement.^16,20^

From an EOR standpoint, the formulations demonstrated three relevant practical implications. The first is the efficient mobilization of residual oil, resulting from the combination of low interfacial tension (IFT) and high interfacial flexibility, which reduces capillary forces and facilitates the displacement of oil trapped within the reservoir pores. The second concerns the improvement of the mobility ratio, achieved through the balance between moderate viscosity and colloidal stability, enabling uniform microemulsion flow during injection. Finally, the third implication relates to the stability of the systems under reservoir conditions and their tolerance to variations in salinity and temperature, as also reported by Aum et al.^40^ and Fukumoto et al..^41^

Although surfactant adsorption onto reservoir rock surfaces is a critical factor influencing chemical loss and the economic feasibility of chemical enhanced oil recovery (EOR) applications,^24,31,49^ this phenomenon was not experimentally evaluated in the present study. The scope of this work was intentionally focused on formulation design, phase behavior, and thermodynamic performance of biobased microemulsion systems. Surfactant adsorption is known to be governed by multiple parameters, including rock mineralogy, surface charge, brine composition, and surfactant molecular structure.^53,54^ In this context, previous studies suggest that biobased surfactants may exhibit lower adsorption tendencies compared to conventional synthetic surfactants, which has been attributed to the presence of specific functional groups and enhanced molecular flexibility that can reduce strong interactions with mineral surfaces.^54,55^ Based on these literature reports, it is expected that the high interfacial mobility and compositional adaptability observed for the proposed biobased formulations could potentially contribute to mitigating surfactant retention in reservoir rocks. However, this hypothesis remains to be quantitatively validated through dedicated adsorption and core-scale experiments, which are planned for future investigations.

Within this framework, the physicochemical properties demonstrated in the present study indicate that the formulated microemulsions are promising candidates for use as injectable fluids in chemical EOR applications, particularly in heterogeneous and high-salinity reservoirs where conventional surfactants typically lose efficiency. Furthermore, the use of renewable raw materials reinforces the alignment of this technology with the principles of sustainability and environmental impact reduction, in accordance with current trends in the petroleum industry.^3,7,16,20^

From a practical implementation standpoint, the scalability and sourcing variability of biobased components are critical factors governing their adoption in EOR operations. Pine oil and coconut oil derivatives are not specialized laboratory materials but well-established agroindustrial commodities, produced at large industrial scales for applications in flotation processes, fragrances, pharmaceuticals, and biofuels.^13,56^ Their industrial production relies on consolidated and geographically diversified supply networks, which reduces risks associated with availability and price volatility, making them economically competitive alternatives to conventional synthetic surfactants.^13,45^

Although natural feedstocks inherently exhibit compositional variability depending on botanical origin, processing methods, and seasonal conditions, recent studies demonstrate that EOR formulations based on renewable oils benefit from broader formulation windows and enhanced interfacial flexibility compared with more rigid petrochemical systems.^17,32^ In this study, the use of wide Winsor IV domains and composition-tolerant microemulsion systems reduces sensitivity to moderate variations in the oil phase, ensuring consistent phase behavior and robust interfacial performance without requiring strict chemical uniformity.^9,17^

This formulation strategy contrasts with polymer-or nanoparticle-assisted systems, whose performance often depends on tightly controlled molecular weight distributions, complex surface functionalization, or dispersion stability that is particularly vulnerable under high-salinity conditions.^31,57^ By exploiting the intrinsic molecular polarity and hydrogen-bonding capacity of naturally functionalized oils, such as the terpenes and terpene alcohols present in pine oil, the proposed biobased microemulsions maintain robustness under realistic supply chain variability.^17^ Consequently, the system offers not only environmental compatibility but also practical viability for large-scale chemical EOR deployment, effectively bridging laboratory-scale performance with industrial sourcing realities and field-scale operability.^13,56^

Recent advances in functional biomaterials and interfacial engineering provide a valuable cross-disciplinary framework for contextualizing the formulation strategy adopted in this study. Research on microstructured and encapsulated systems has demonstrated that adaptive interfacial architectures, capable of reorganizing under external stimuli while preserving structural integrity, are critical for achieving controlled transport, stability, and performance in complex environments.^58,59^ These principles closely mirror the physicochemical requirements of effective EOR formulations, where interfacial flexibility, robustness against compositional perturbations, and sustained functionality under dynamic flow conditions are essential.

In this context, the biobased Winsor IV microemulsions developed herein can be viewed as molecularly adaptive systems, in which naturally functionalized oil phases and surfactants self-assemble into resilient interfacial films without reliance on encapsulation or solid scaffolds. By achieving stability and transport efficiency through intrinsic molecular design rather than structural confinement, the proposed formulations align with emerging paradigms in soft matter and biomaterials science, broadening their technological relevance beyond conventional chemical EOR and reinforcing their potential for translation across energy and materials engineering applications.

Recent state-of-the-art advances in chemical EOR have been predominantly driven by increasingly complex multicomponent formulations, including polymer-enhanced surfactant systems, alkali–surfactant–polymer (ASP) formulations, and hybrid approaches incorporating nanoparticles to improve phase stability, mobility control, and tolerance to salinity and temperature.^5,30,60,61^ While often effective, these systems introduce significant operational and economic challenges, such as polymer degradation under shear and thermal stress, nanoparticle aggregation, injectivity impairment, and increased costs associated with synthesis, functionalization, and produced-water treatment.^31,57^

In contrast, the present study demonstrates that comparable interfacial efficiency and phase robustness can be achieved through rational formulation design, without the need for external polymers or nanoparticles. By exploiting the intrinsic polarity, molecular flexibility, and hydrogen-bonding capacity of naturally functionalized oil phases (pine oil) and biobased surfactants (saponified coconut oil), the proposed system spontaneously expands the single-phase Winsor IV domain and attains low interfacial tensions with favorable pseudoplastic rheology. This molecular-level “green” design effectively mimics the key performance attributes of complex surfactant–polymer systems, while avoiding their susceptibility to shear- and thermally induced degradation.^9^ Moreover, the use of renewable agroindustrial feedstocks reduces formulation complexity, environmental impact, and logistical demands, offering a simplified yet robust alternative to polymer-based and hybrid chemical EOR technologies. Rather than representing an incremental green substitution, the biobased Winsor IV microemulsions proposed herein position themselves as a competitive and environmentally viable strategy capable of meeting the critical interfacial, rheological, and stability requirements traditionally addressed through increased chemical complexity.

In summary, biobased formulations that achieve a complete “puzzle-like” balance, characterized by well-tuned interactions among the surfactant, cosurfactant, nonpolar phase, and polar phase, are more likely to maximize the thermodynamic and kinetic stability of the microemulsion, resulting in superior displacement efficiency under reservoir conditions.

## 4 Conclusion

The microemulsion systems investigated in this study demonstrated the technical feasibility of formulating Winsor IV structures with low interfacial tension, moderate viscosity, and broad single-phase regions, which are essential characteristics for EOR applications. Formulations containing functionalized oils, such as pine oil, combined with natural surfactants like saponified coconut oil, exhibited superior phase stability and interfacial performance compared to systems based on purely aromatic oils such as toluene.

The composition of the polar phase also had a significant impact. The addition of salt (Formulations F6 and F7) slightly reduced the Winsor IV region but maintained sufficiently low interfacial tensions, which are essential for capillary-driven oil mobilization. Glycerol (Formulation F3) improved the kinetic stability by delaying phase separation, although its effect required careful optimization to prevent compromising the solubilization capacity.

The “puzzle analogy” proved useful for interpreting the cooperative interactions among the formulation components. Optimal performance was achieved when the chemical nature of the nonpolar phase, surfactant, cosurfactant, and polar phase was harmonized to promote interfacial flexibility and stabilization.

Overall, the results suggest that environmentally friendly microemulsion systems, designed from renewable raw materials and optimized through careful component selection, represent a promising pathway toward sustainable and efficient oil recovery strategies, aligning with the principles of the United Nations Sustainable Development Goals.

## References

[1] Hon V. Y., Saaid I. M., Chai I. C. H., Fauzi N. A. A. M., Deguillard E., Van Male J., Handgraaf J.-W. (2022). Microemulsion
Interface Model for Chemical Enhanced Oil Recovery Design. J. Pet. Sci. Eng..

[2] Mariyate J., Bera A. (2022). A Critical Review on Selection of
Microemulsions or Nanoemulsions
for Enhanced Oil Recovery. J. Mol. Liq..

[3] Santos L. B. L., Silva A. C. M., Pereira K. R. O., Moraes C., Leiras Gomes A. C., Santos J. P. L., Simonelli G., Santos L. C. L. (2023). Microemulsions
Stabilized with Nanoparticles for EOR: A Review. J. Mol. Liq..

[4] Mahboob A., Kalam S., Kamal M. S., Hussain S. M. S., Solling T. (2022). EOR Perspective
of Microemulsions: A Review. J. Pet. Sci. Eng..

[5] Ray D., Jangid L., Joshi D., Prakash S., Ojha K., Manor O., Mandal A. (2024). Formulation
of Polymer-Augmented
Surfactant-Based Oil–Water Microemulsions for Application in
Enhanced Oil Recovery. ACS Omega.

[6] Zhang G., Zheng Y., Tian F., Liu H., Lu X., Yi X., Wang Z. (2023). Performance of Extended
Surfactant and Its Mixture
with Betaine Surfactant for Enhanced Oil Recovery in Sandstone Reservoirs. J. Mol. Liq..

[7] Dutta S. J., Neog D. (2025). Sustaining Heavy Crude Oil Production
with Chemical EOR: A Comparative
Study. Chem. Thermodyn. Therm. Anal..

[8] Castro
Dantas T. N., Viana F. F., de Souza T. T. C., Dantas
Neto A. A., Aum P. T. P. (2021). Study of Single-Phase Polymer–Alkaline–Microemulsion
Flooding for Enhancing Oil Recovery in Sandstone Reservoirs. Fuel.

[9] Pal N., Alzahid Y., AlSofi A. M., Ali M., Yekeen N., Hoteit H. (2023). An Experimental Workflow to Assess
the Applicability
of Microemulsions for Conformance Improvement in Oil-Bearing Reservoir. Heliyon.

[10] Zhou Y., Yin D., Wang D., Zhang C., Yang Z. (2020). Experiment Investigation
of Microemulsion Enhanced Oil Recovery in Low Permeability Reservoir. J. Mater. Res. Technol..

[11] Pal N., Alzahid Y., AlSofi A. M., Ali M., Hoteit H. (2023). Review on
Microemulsions for Conformance Improvement Technology: Fundamentals,
Design Considerations, and Perspectives. Energy
Fuels.

[12] Nadir N., Shahruddin S., Othman J. (2022). Surfactant Evaluation for Enhanced
Oil Recovery: Phase Behavior and Interfacial Tension. Open Chem..

[13] Abdurrahman M., Kamal M. S., Ramadhan R., Rita N., Al-Nakhli A. R., Al-Mubarak S. A., Mahmoud M. (2023). Ecofriendly Natural Surfactants in
the Oil and Gas Industry: A Comprehensive Review. ACS Omega.

[14] Quintella C. M. (2025). Environmental
Protection in Enhanced Oil Recovery and Its Waste and Effluents Treatment:
A Critical Patent-Based Review of BRICS and Non-BRICS (2004–2023). Sustainability.

[15] Rodrigues P. D., Quintella C. M., Nicoleti J. L., Carvalho E. B., Medeiros A. C. G., Ramos-de-Souza E., Santos E. S., Vasconcelos A. C., Moura J. D. (2025). Investigation of
the Synergistic Effects of Different
Salts in Smart Water Injection Fluids on Oil–Brine Interfacial
Tension. ACS Omega.

[16] Pal N., Kumar S., Bera A., Mandal A. (2019). Phase Behaviour and
Characterization of Microemulsion Stabilized by a Novel Synthesized
Surfactant: Implications for Enhanced Oil Recovery. Fuel.

[17] dos
Santos A. V., Dos Santos V. F. B., Fernandes V. C., Figueiredo S. A., Grosso C., Delerue-Matos C., Simonelli G., Santos L. C. L. (2025). Eco-Friendly Cleaning Agent Applied
for Ex Situ Remediation of Beach Sand Contaminated with Crude Oil. Environ. Geochem. Health.

[18] Mehrjoo, H. ; Riazi, M. ; Norouzi-Apourvari, S. A Comprehensive Review on the Use of Eco-Friendly Surfactants in Oil Industry. In Green Sustainable Process for Chemical and Environmental Engineering and Science; Elsevier: Amsterdam, 2021; pp 357–399 10.1016/B978-0-12-821931-7.00009-2.

[19] Karambeigi M. S., Nasiri M., Haghighi
Asl A., Emadi M. A. (2016). Enhanced Oil Recovery
in High Temperature Carbonates Using Microemulsions Formulated with
a New Hydrophobic Component. J. Ind. Eng. Chem..

[20] Qin T., Javanbakht G., Goual L., Piri M., Towler B. (2017). Microemulsion-Enhanced
Displacement of Oil in Porous Media Containing Carbonate Cements. Colloids Surf., A.

[21] Pal N., Alzahid Y., AlSofi A. M., Ali M., Zhang X., Hoteit H. (2023). Experimental Evaluation of Surfactant-Stabilized
Microemulsions
for Application in Reservoir Conformance Improvement Technology. J. Mol. Liq..

[22] Xiao L., Hou J., Sun J., Yang Y. (2025). Phase Behavior
of EOR-Oriented Dilutable
Single-Phase Microemulsions. Fuel.

[23] Ramesh
Dadi N., Kumar Maurya N., Gupta P. (2024). Advancing Foam EOR: A Comprehensive
Examination of Key Parameters and Mechanisms from Surfactants to Nanoparticles. J. Mol. Liq..

[24] Bera A., Kumar T., Ojha K., Mandal A. (2014). Screening
of Microemulsion
Properties for Application in Enhanced Oil Recovery. Fuel.

[25] Santanna V. C., Curbelo F. D. S., Castro Dantas T. N., Dantas Neto A. A., Albuquerque H. S., Garnica A. I. C. (2009). Microemulsion Flooding for Enhanced
Oil Recovery. J. Pet. Sci. Eng..

[26] Castro
Dantas T. N. d., de Souza T. T. C., Rodrigues M. A. F., Dantas Neto A. A., Aum P. T. P. (2022). Experimental
Study of Combined Microemulsion/Brine Flooding to EOR in Carbonate
Reservoirs. J. Dispersion Sci. Technol..

[27] Saw R. K., Bhadiyadra C., Jangid L., Mandal A. (2025). Enhanced Oil Recovery
Analysis of Green Nanoemulsion Flooding for Sandstone Reservoirs. Energy Fuels.

[28] Jeirani Z., Mohamed Jan B., Si Ali B., See C. H., Saphanuchart W. (2014). Pre-prepared
Microemulsion Flooding in Enhanced Oil Recovery: A Review. Pet. Sci. Technol..

[29] de
Oliveira G. V. B., de Araújo J. D.
C., Lourenço M. C. M., de Freitas A. P. T. A., Rodrigues M. A. F., Dantas T. N. C., Wanderley Neto A. O., Haas D. A. (2023). Study on Steam and
Microemulsion Alternate Injection for Enhanced Productivity in Heavy
Oil Fields. Energy Fuels.

[30] Tliba L., Edokali M., Mehrabi M., Glover P. W. J., Menzel R., Hassanpour A. (2025). Enhancing
Oil Recovery with Shape-Modified Silica Nanoparticles:
Efficiency in Oil-Wet Sandstone Reservoirs via Imbibition and Micromodel
Approaches. Energy Fuels.

[31] Mumbere W., Sagala F., Gupta U., Bbosa D. (2025). Reservoir Potential
Unlocked: Synergies between Low-Salinity Water Flooding, Nanoparticles,
and Surfactants. ACS Omega.

[32] Ferreira G. F. D., Souza D. R. Q., Lima R., Lobato A. K. C. L., Silva A. C. M., Santos L. C. L. (2018). Novel Glycerin-Based
Microemulsion
Formulation for Enhanced Oil Recovery. J. Pet.
Sci. Eng..

[33] Jeirani Z., Mohamed Jan B., Si Ali B., Noor I. M., See C. H., Saphanuchart W. (2013). Formulation, Optimization and Application of Triglyceride
Microemulsion in Enhanced Oil Recovery. Ind.
Crops Prod..

[34] Surfactants: Fundamentals and Applications in the Petroleum Industry; Schramm, L. L. , Ed.; Cambridge University Press, 2000 10.1017/CBO9780511524844.

[35] Fanun M. (2010). Properties
of Microemulsions with Mixed Nonionic Surfactants and Citrus Oil. Colloids Surf., A.

[36] Araújo C. R. B.
d., Silva D. C., Arruda G. M., Rodrigues M. A. F., Wanderley Neto A. D. O. (2021). Removal
of Oil from Sandstone Rocks
by Solid–Liquid Extraction Using Oil Phase-Free Microemulsion
Systems. J. Environ. Chem. Eng..

[37] Rezaie A., Ghasemi H., Eslami F. (2023). An In-Depth Investigation
of the
Impact of Salt Nature on the Formulation of Microemulsion Systems. Sci. Rep..

[38] Moulik S. P., Paul B. K. (1998). Structure, Dynamics and Transport
Properties of Microemulsions. Adv. Colloid Interface
Sci..

[39] Hu H., Zhang Q., Tian M., Li Y., Han X., Guo R. (2024). Review: Microemulsions
for the Sustainable Development of EOR. Sustainability.

[40] Aum Y. K. P. G., Aum P. T. P., Silva D. N. N. D., Cavalcante L. A., Barros Neto E. L., Castro Dantas T. N. (2023). Characterization of Oil-in-Water
Microemulsions Based on Ethoxylated Surfactant for Paraffinic Deposits
Removal. Fuel.

[41] Fukumoto A., Dalmazzone C., Frot D., Barré L., Noïk C. (2016). Investigation on Physical Properties and Morphologies
of Microemulsions Formed with Sodium Dodecyl Benzenesulfonate, Isobutanol,
Brine, and Decane Using Several Experimental Techniques. Energy Fuels.

[42] Sousa R. P. F. d., Braga G. S., Silva R. R., Leal G. L. R., Freitas J. C. O., Madera V. S., Garnica A. I. C., Curbelo F. D. S. (2021). Formulation and
Study of an Environmentally Friendly Microemulsion-Based Drilling
Fluid (O/W) with Pine Oil. Energies.

[43] Mariyate J., Bera A. (2021). Recent Progresses of
Microemulsions-Based Nanofluids as a Potential
Tool for Enhanced Oil Recovery. Fuel.

[44] Franco C. A., Giraldo L. J., Candela C. H., Bernal K. M., Villamil F., Montes D., Cortés F. B. (2020). Design and Tuning of
Nanofluids Applied to Chemical Enhanced Oil Recovery Based on the
Surfactant–Nanoparticle–Brine Interaction: From Laboratory
Experiments to Oil Field Application. Nanomaterials.

[45] Queiroz
Neto J. C. d., Remboski T. A., Leal G. L. R., Pessoa M. E. A., Freitas J. C. O., Curbelo F. D. S. (2025). Microemulsions
and Nanoparticles: The Sustainable Future of Drilling Fluids in Oil
Exploration. Rev. Gest. Sustent. Ambient..

[46] Li D., Wang Y., Liang S., Bai B., Zhang C., Xu N., Shi W., Ding W., Zhang Y. (2024). Synthesizing Dendritic
Mesoporous Silica Nanoparticles to Stabilize Pickering Emulsions at
High Salinity and Temperature Reservoirs. Colloids
Surf., A.

[47] Mandal, A. ; Ojha, K. Enhanced Oil Recovery: Mechanisms, Technologies and Feasibility Analyses; CRC Press: Boca Raton, FL, 2024 10.1201/9781003098850.

[48] Singh P. K., Joshi D., Mandal A., Pal N. (2025). Silica Nanoparticle-Stabilized
Anionic Surfactant Microemulsions: Characterization, Technical Evaluation,
and Core-Flooding Studies for Enhanced Oil Recovery. Energy Fuels.

[49] Kaushik A., Joshi D., Saw R. K., Rathi K. B., Mitra S., Mandal A. (2024). Formation and Characterization
of Nanoparticle Assisted
Surfactant Stabilized Oil-in-Water Nanoemulsions for Application in
Enhanced Oil Recovery. Fuel.

[50] Li Y., Yang M., He T., Chen J. (2025). Study on Rapid Construction
of Microemulsion System Based on EACN of Crude Oil Measured by the
Direct Method. Petroleum.

[51] Nguyen T. T., Morgan C., Poindexter L., Fernandez J. (2019). Application
of the Hydrophilic–Lipophilic Deviation Concept to Surfactant
Characterization and Surfactant Selection for Enhanced Oil Recovery. J. Surfactants Deterg..

[52] Leng K., Guan B., Liu W., Jiang C., Cong S., Peng B., Tao Y. (2024). Advance of Microemulsion and Application
for Enhanced Oil Recovery. Nanomaterials.

[53] Wesson, L. L. ; Harwell, J. H. Surfactant Adsorption in Porous Media. In Surfactants: Fundamentals and Applications in the Petroleum Industry; Schramm, L. L. , Ed.; Cambridge University Press: Cambridge, U.K., 2000; pp 121–158 10.1017/CBO9780511524844.

[54] Hama S. M., Manshad A. K., Ali J. A. (2023). Review of the Application of Natural
Surfactants in Enhanced Oil Recovery: State-of-the-Art and Perspectives. Energy Fuels.

[55] Adenutsi C. D., Turkson J. N., Sokama-Neuyam Y. A. (2023). Review
on Potential Application of
Saponin-Based Natural Surfactants for Green Chemical Enhanced Oil
Recovery. Energy Fuels.

[56] Sharma K., Toor S. S., Brandão J., Pedersen T. H., Rosendahl L. A. (2021). Optimized
Conversion of Waste Cooking Oil into Ecofriendly Bio-Based Polymeric
Surfactant: A Solution for Enhanced Oil Recovery and Green Fuel Compatibility. J. Clean. Prod..

[57] Lao J., Cheng H., Wang Y., Song H. (2024). Micro/Nanoparticular
Flow in Porous Media for Enhanced Oil Recovery: A Review. Energies.

[58] Zheng M., Wu Z., Liu T., Yan S., Li X., Jiang G., Yang G., Du Y., Zhang Y. (2026). Optimization of Microcrack
Control and Performance in Deepwater Well Cementing with Microencapsulated
Phase Change Materials. Geoenergy Sci. Eng..

[59] Wang Y., Fang Y., Hu X., Sun Y., Li H., Xia Y. (2025). Predicting the Foamability of *N*-Acyl Amino Acid
Surfactants via Noncovalent Interactions. Colloids
Surf., A.

[60] Sagandykova D., Shakeel M., Pourafshary P. (2024). Combining Thermal Effect and Mobility
Control Mechanism to Reduce Water Cut in a Sandstone Reservoir in
Kazakhstan. Polymers.

[61] Ahmadi B., Sahraei E., Mohammadi A. H. (2025). Investigation
of the Synergistic
Effects of SiO_2_ and Al_2_O_3_ Nanoparticles
with SDS and CTAB Surfactants on the Stability and Improving Phase
Behavior of Water–Oil Emulsions. Colloids
Surf., A.

